# Tumor bacterial markers diagnose the initiation and four stages of colorectal cancer

**DOI:** 10.3389/fcimb.2023.1123544

**Published:** 2023-03-13

**Authors:** Ping Cai, Jinbo Xiong, Haonan Sha, Xiaoyu Dai, Jiaqi Lu

**Affiliations:** ^1^ Ningbo Second Hospital, Ningbo, China; ^2^ Ningbo Institute of Life and Health Industry, University of Chinese Academy of Sciences, Ningbo, China; ^3^ State Key Laboratory for Managing Biotic and Chemical Threats to the Quality and Safety of Agro-products, Ningbo University, Ningbo, China; ^4^ Key Laboratory of Marine Biotechnology of Zhejiang Province, School of Marine Sciences, Ningbo University, Ningbo, China; ^5^ Zhejiang KinGene Bio-technology Co., Ltd, Ningbo, China

**Keywords:** colorectal cancer (CRC) stage, CRC-associated taxa, average variation degree, occupancy and specificity, CRC-stage discriminatory taxa, diagnosis model

## Abstract

Increasing evidence has supported dysbiosis in the faecal microbiome along control-adenoma-carcinoma sequence. In contrast, the data is lacking for *in situ* tumor bacterial community over colorectal cancer (CRC) progression, resulting in the uncertainties of identifying CRC-associated taxa and diagnosing the sequential CRC stages. Through comprehensive collection of benign polyps (BP, *N* = 45) and the tumors (*N* = 50) over the four CRC stages, we explored the dynamics of bacterial communities over CRC progression using amplicons sequencing. Canceration was the primarily factor governing the bacterial community, followed by the CRC stages. Besides confirming known CRC-associated taxa using differential abundance, we identified new CRC driver species based on their keystone features in NetShift, including *Porphyromonas endodontalis*, *Ruminococcus torques* and *Odoribacter splanchnicus.* Tumor environments were less selective for stable core community, resulting in heterogeneity in bacterial communities over CRC progression, as supported by higher average variation degree, lower occupancy and specificity compared with BP. Intriguingly, tumors could recruit beneficial taxa antagonizing CRC-associated pathogens at CRC initiation, a pattern known as “cry-for-help”. By distinguishing age- from CRC stage-associated taxa, the top 15 CRC stage-discriminatory taxa contributed an overall 87.4% accuracy in diagnosing BP and each CRC stage, in which no CRC patients were falsely diagnosed as BP. The accuracy of diagnosis model was unbiased by human age and gender. Collectively, our findings provide new CRC-associated taxa and updated interpretations for CRC carcinogenesis from an ecological perspective. Moving beyond stratifying case-control, the CRC-stage discriminatory taxa could add the diagnosis of BP and the four CRC stages, especially the patients with poor pathological feature and un-reproducibility between two observers.

## Introduction

Colorectal cancer (CRC) is the third most prevalent cancer, which ranks the second in terms of mortality globally (Arnold et al., 2017; Cai and Liu, 2021). Although the incidence and mortality rates of CRC are decreasing in recent years, CRC is still one of the most life-threatening cancers and advanced CRC remains an incurable disease at metastatic stages, 5-year survival rate of 13% compared to 90% when diagnosed at an initial stage (Bray et al., 2018; Montalban-Arques and Scharl, 2019). The trend in younger patients, along with the continued burden, highlight the need for early detection of CRC that alleviates the incidence of metastatic CRC (Cheng et al., 2020). In particular, the clinical trials of patients are intimately associated with the CRC stages, thus there is an urgent requirement for accurately diagnosing the stages of CRC.

Over the past decades, the Tumour, Nodes, and Metastasis (TNM) staging system has contributed the cornerstone for the management of CRC patients. However, some problems have occurred with the TNM system, such as increasing complexity of CRC, poor clinical evidence, tumor deposits, and un-reproducibility between different observers (Quirke et al., 2007), which in turn confuse the accuracy of identified CRC stage and subsequent therapy. It is now widely recognized that the microbes contribute indispensable roles in host health and gastrointestinal tumor progression (Marchesi et al., 2016; Xiong, 2018; Guo et al., 2022). In accordance, intensive studies have shown dysbiosis (shift in gut commensal microbiota toward opportunistic pathogens) in the gut microbiota in CRC patients compared with healthy controls. Going forward, CRC-associated taxa have been identified for distinguishing healthy from colorectal adenomas, and CRC individuals (Shah et al., 2018; Wu et al., 2021; Coker et al., 2022). However, few studies have explored the dynamics of microbial communities over CRC progression. As a result, it is uncertain whether microbial taxa are indicative of each CRC stage, rather than just stratify case from control cohorts. However, this information is fundamental to establish CRC stage-dependent clinical trials.

Accumulating evidence depicts that the gut microbiota is an important etiological element in CRC initiation, progression, and metastasis (Kong et al., 2019; Cheng et al., 2020; Mizutani et al., 2020; Li et al., 2022). By this logic, identification of bacteria involved in CRC progression could provide new targets for CRC diagnosis and prevention (Montalban-Arques and Scharl, 2019; Fong et al., 2020). Indeed, case studies have proposed that the occurrence of CRC is attributed to the enrichment of *Fusobacterium nucleatum* (encoding FadA) (Flanagan et al., 2014), *Bacteroides fragilis* and *Escherichia coli* hosting polyketide synthase (pks) islands (Feng et al., 2015), *Clostridium symbiosum* (Xie et al., 2017), or *Parvimonas micra* (Löwenmark et al., 2020), respectively. It should be noted that the identification of “driver” taxa is generally based on their significant enrichment in CRC patients compared to healthy controls, which ignores the CRC stage (Flanagan et al., 2014; Löwenmark et al., 2020). In particular, a “driver” taxon is attributed to strong biotic interactions, rather than its sheer abundances, though this does not rule out that some “driver” taxa are numerically abundant (Dai et al., 2018). Recently, NetShift approach has been developed to quantify major changes in associations of each constituent taxon between healthy and diseased networks, in which the importance of a single species between health states can be calculated based on its topological features (Kuntal et al., 2019). By this logic, taxa that increase in their importance in the network of CRC patients could be the “driver” taxa, moving beyond enriched abundance. Furthermore, according to the “driver-passenger” model, the CRC driver taxa could be superseded by “passenger” bacteria that are better adapted to the conditions in and around carcinoma cells, thereby outcompeting the initial driver species (Feng et al., 2015; Bridges et al., 2019). For these reasons, a systematic analysis of CRC-associated bacteria along CRC progression is required from an ecological prospective, rather than case-control study.

In spite of a growing body of evidence with regard to the dysbiosis in gut microbiota in CRC patients, data on the association between *in situ* tumor microbial dynamics over CRC progression is lacking. The gut microbiotas are significantly varied as over human lifetime (Falony et al., 2016; Greenhalgh et al., 2016; Ghosh et al., 2020), thus a key challenge is to distinguish the alterations in microbial assembly over CRC progression from these as human aged. Additionally, faecal microbiota only partially mirrors mucosal microbiota in CRC, with low correlations between paired faecal and mucosal samples (Flemer et al., 2017). In this regard, the deployment of faecal microbes for mirroring tumor microbiota could bias the identification of CRC-associated bacteria. To overcome above obstacles, we explored the dynamics of bacterial communities in tumor tissues along the four CRC stages, and benign polyps as controls. We attempted to address the following concerns: (1) exploring the dynamics of bacterial communities over CRC progression, (2) identifying CRC-associated bacteria based on biotic interactions, (3) screening biomarkers for diagnosing each CRC stage, irrespective of host age.

## Materials and methods

### Experimental design and sample collection

Subjects underwent standard colonoscopy examinations at Hwa Mei Hospital in Ningbo City, China, were recruited to the study. The patients were selected based on the following criteria: no complicating diseases (such as chronic bowel disease, diabetes, and hypertension); no family history of CRC and recurrence in CRC patients, no use of antibiotic in the month prior to surgery. Written informed consent was obtained from the volunteers to utilize their tissue samples. All volunteers were categorized into a different group based on the histopathological features in the TNM staging system of malignant tumors after surgery. The samples with uncertain TNM stage (e.g., poor clinical evidence, tumor deposits, un-reproducibility between two observers) were excluded in the analysis. Based on these selective criterions, 95 subjects (aged 21–89 years, 70 males and 25 females) were enrolled in the analysis from 120 volunteers, in which included 45 BPs and 50 tumors over the four CRC stages. The general information (age, gender) and clinical data (body mass index (BMI), carcinoembryonic antigen (CEA), TNM stage) are summarized in **Table S1** and **Figure S1**. All tissue samples were stored at −80°C until further processing. We want to point out that no tissue samples can be obtained from healthy individuals, thus benign polyps (BPs) were served as controls.

### DNA extraction, amplification, and sequencing of the 16S rDNA genes

Tumor or BP tissue (1 gram) were homogenized with four volumes (weight/volume) of phosphate buffer solution (PBS, pH = 7.4) and centrifuged at 4000 rpm for 5 min. To collect microbial biomasses, the supernatant was transferred and centrifuged at 12000 rpm for 10 min at 4°C. DNA was extracted using the FAST DNA Spin kit (MoBio Laboratories, Carlsbad, CA, USA) following the manufacturer’s protocols. The concentration and purity of DNA extracts were evaluated by using a NanoDrop ND-2000 spectrophotometer (NanoDrop Technologies, Wilmington, USA). The V3–V4 regions of bacterial 16S rDNA genes were amplified by using the primer pair: 341F (5’-CCTACGGGNGGCWGCAG-3’) and 806R (5’-GGACTACHVGGGTWT- CTAAT-3’) (Takahashi et al., 2014). To minimize PCR induced biases, each sample was amplified in triplicates as follows: 25 cycles of denaturation at 95°C for 30 s, annealing at 55°C for 30 s, and extension at 72°C for 45 s, with a final elongation step of 72°C for 10 min in 30 μL PCR reaction system. Every triplicate amplicons from each sample were pooled and purified using a PCR fragment purification kit. The concentrations of purified products were detected using a PicoGreen-iT dsDNA Assay Kit (Invitrogen, Carlsbad, USA). Equimolar amounts of amplicons for each sample were pooled, and sequenced on a single run using an Illumina MiSeq platform (Illumina, San Diego, USA), producing 2 × 300 bp paired-end reads.

### Processing of Illumina sequencing data

The FASTQ format data were analyzed by the Quantitative Insights into Microbial Ecology 2 (QIIME 2) pipeline (Bolyen et al., 2019). In short, the raw sequences were processed using the Divisive Amplicon Denoising Algorithm 2 (DADA2) that could obtain reads with a single-nucleotide difference (Callahan et al., 2016), known as amplicon sequence variants (ASVs). Primers were screened and removed. Filtered reads were then de-replicated and de-noised using DADA2 with default parameters. Then, paired-end sequences were merged, and chimeras were identified and removed using Usearch (version 11.0.667) and the “uchime2_ref” command. Reads were truncated at the quality control score of 20. Taxonomic assignment of ASVs was performed based on the SILVA v138 16S database (Quast et al., 2012). ASVs classified as Mitochondria, Chloroplast, Archaea, and Eukaryota in origin were removed from the bacterial community. Only ASVs detected with a minimum of three samples were included. Finally, to adjust unequal sequencing depth, all samples were rarefied to the same sequencing depth in downstream analysis. After filtering and rarefaction to 14,221 reads per sample, a total of 3601 ASVs were included in the final analyses.

### Diagnosis model of CRC stages

To identify bio-indicators for quantitatively diagnosing the stages of CRC, we created a classification model using random forests (RF), a robust machine-learning algorithm for classification and regression that is suitable for microbial population data (Liaw and Wiener, 2002). Given that the structures of bacterial community were highly temporal dynamics over human ontogeny, we first determined host age-discriminatory lineages (when bacterial taxonomic level is undefined, namely, bacterial phylum, class, order, family, genus, or ASV) across the BP controls. The relative abundances of all lineages in BP were regressed against corresponding host age using default parameters. The 10-fold cross-validation function was implemented to identify the minimal number of top-ranking age-discriminatory lineages, which only contained the most important variables based on the cross-validation curve (Fushiki, 2011). To rule out the ontogenic effects on bacterial community, the age-discriminatory lineages were excluded from the dataset. Then, the relative abundances of all lineages were classified into corresponding stages, that is, BPs and the four CRC stages (**Table 1**). The RF model was repeated to identify the top CRC stage-discriminatory lineages. After this optimization, the identified CRC stage-discriminatory lineages were employed as dependent variables for diagnosing BP and CRC stages. A consistency between observed and diagnosed category was termed a correct diagnosis; otherwise, the classification was termed as a false diagnosis. To acquire finer taxonomic information for the CRC stage-discriminatory ASVs, we manually identified their species classification by aligning their representative sequences in the basic local alignment searching tool (BLAST, https://blast.ncbi.nlm.nih.gov/Blast.cgi).

**Table 1:** The predicted accuracy based on profiles of colorectal cancer (CRC) stage-discriminatory lineages at bacterial phylum, class, order, family, genus or ASV level, respectively.

Taxonomy	Observed	Predicted					Overall accuracy
		T1	T2	T3	T4	BP	
Phylum	T1	**3**	1	2	3	0	(59/95) 62.1%
	T2	0	**1**	8	0	4	
	T3	1	3	**12**	1	5	
	T4	1	3	2	**0**	0	
	BP	0	0	2	0	**43**	
Class	T1	**2**	2	1	0	4	(61/95) 64.2%
	T2	1	**2**	6	0	4	
	T3	2	4	**11**	1	4	
	T4	0	2	3	**1**	0	
	BP	0	0	0	0	**45**	
Order	T1	**1**	3	2	0	3	(61/95) 64.2%
	T2	0	**0**	10	0	3	
	T3	1	2	**14**	0	5	
	T4	2	0	2	**1**	1	
	BP	0	0	0	0	**45**	
Family	T1	**0**	1	2	4	2	(63/95) 66.3%
	T2	0	**2**	8	0	3	
	T3	1	2	**14**	0	5	
	T4	0	4	0	**2**	0	
	BP	0	0	0	0	**45**	
Genus	T1	**1**	0	2	5	1	(60/95) 70.6%
	T2	0	**0**	11	0	2	
	T3	1	3	**16**	0	2	
	T4	0	0	2	**0**	4	
	BP	0	0	2	0	**43**	
ASV	T1	**8**	0	1	0	0	(83/95) 87.4%
	T2	1	**8**	4	0	0	
	T3	0	2	**17**	3	0	
	T4	0	0	0	**6**	0	
	BP	0	0	1	0	**44**	

BP, Benign polyps, T1, T2, T3 and T4 were the four CRC stages.

For a given sample, the consistency between observed and diagnosed category was termed as a correct diagnosis, otherwise it was termed as a false diagnosis. Bold values represent the numbers of correct diagnoses.

### Statistical analysis

The following analyses were performed in R 3.6.3, unless otherwise stated (http://www.R-project.org/). Alpha diversity of bacterial community was compared among CRC stages using one-way analysis of variance (ANOVA). Canonical analysis of principal coordinates (CAP) and non-parametric multivariate analysis of variance (NPMANOVA) were used to assess the differences in bacterial communities along CRC stages based on Bray-Curtis distances (Anderson and Willis, 2003). Statistical differences in beta-diversity between health status, age, and gender were calculated using perMANOVA with the adonis2 function in vegan package (Oksanen et al., 2018). Bacterial community stability was evaluated by average variation degree (AVD). A lower AVD value indicates higher stability (Xun et al., 2021). An UpSet plot was used to display the number of shared and unique bacterial ASVs among groups (Lex et al., 2014). The 500 most abundant ASVs were selected from the bacterial communities in each group, specificity and occupancy were calculated as described previously (Gweon et al., 2021). Specificity is the mean abundance of species (S) in the samples within a group; and occupancy is the relative frequency of occurrence of S in the samples within a group. The two metrics (specificity and occupancy) were used as the axes in SPEC-OCCU biplot (Dufrêne and Legendre, 1997; Gweon et al., 2021). CRC driver taxa were screened based on their “Neighbor Shift (NESH) Score” and node size using NetShift. NESH is a neighbor shift score, which represents directional changes in individual node associations (Kuntal et al., 2019).

## Results

### General and clinical information

Ages of the 50 CRC patients ranged from 30 to 89 years, in which 7 patients (2 individuals were T2, and 5 patients were T3 stage) were younger than 40 years. This pattern reinforced the trend to younger CRC patients. The numbers of male patients were consistently higher (*P* = 0.0105, paired t test fixed CRC stage) than female patients over each CRC stage (**Table S1**). As expected, CEA values linearly increased along CRC severity (**Figure S1A**), while no significant differences in BMI among the four CRC stages (**Figure S1B**).

### Differed microbiotas along CRC stages and BPs

Sequencing yielded a total of 12,128,782 (mean ± standard deviation, 81710 ± 47466) raw reads from the enrolled 95 samples. Rarefaction curves indicated sufficient sequencing depth was achieved (**Figure S2**), thus enabled us to compare diversity among groups. The Firmicutes, Bacteroidota, and Proteobacteria were the dominant bacterial phyla in BPs and tumor tissues, albeit difference in their relative abundances (**Figure S3A**). This composition was analogous to the composition of human gut microbiota. At the finer bacterial genus level, genera of *Collinsella*, *Parvimonas*, *Ruminococcus*, and *Bifidobacterium* significantly enriched in the tumors compared with BP, while the relative abundance of *Faecalibacterium* exhibited the opposing trend (**Figure S3B**).

**Figure 1:**
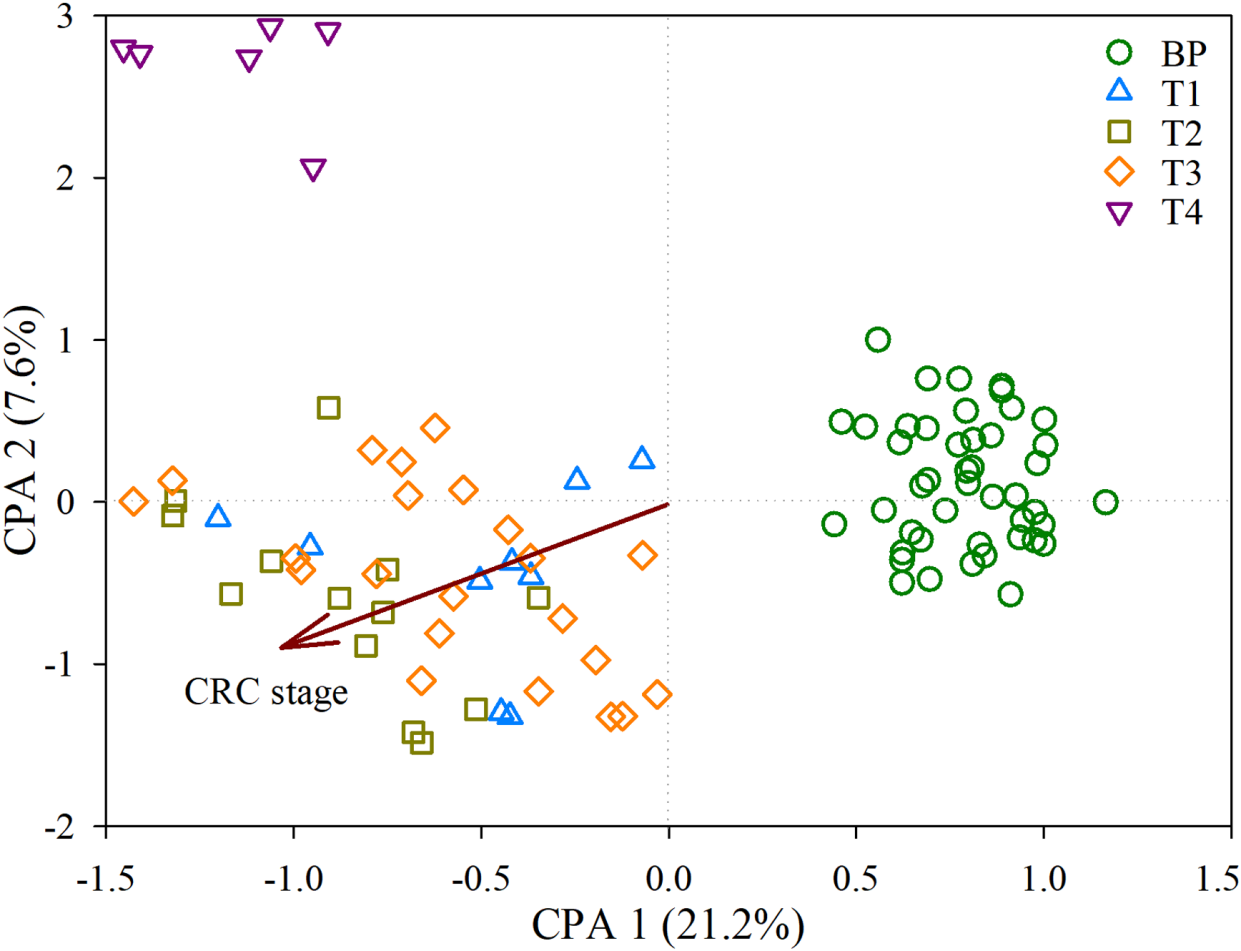
Constrained analysis of principal coordinates (CAP) depicting the effects of CRC stage on the bacterial communities derived from the distance matrix. Samples were coded by benign polyps (BP, here is controls) and along the four colorectal cancer (CRC) stages, T1, T2, T3 and T4.

**Figure 2:**
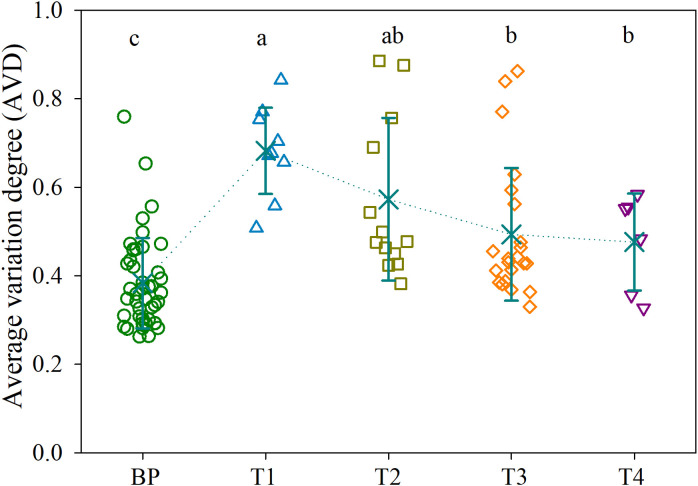
Average variation degree (AVD) for the bacterial communities in BP and along the four CRC stages. Different lowercase letters indicate significant differences among groups using one-way analysis of variance (ANOVA) with significant level of *P* < 0.05. Refer to **Figure 1** for abbreviations.

There were no significant differences in the diversity of bacterial communities among BP and the tumors, as supported by both the Shannon diversity and Phylogenetic diversity (**Figure S4**). However, the CAP biplot demonstrated clear separation of bacterial communities between BP and along the four CRC stages (T1, T2, T3 and T4), in which CRC stage was imposed as a conditional factor. Overall, the bacterial communities were more dissimilar between BP and tumors than along the four CRC stages (**Figure 1**). These patterns were further corroborated by a comparison of the similarity between groups; the structures of bacterial community differed significantly (*P* < 0.05) between each paired groups, with the exception of T1 vs. T3 (**Table S2**). Furthermore, parametric permutational multivariate analysis of variance (perMANOVA) revealed that CRC stage and human age respectively constrained 8.4% (*P* < 0.001) and 2.1% (*P* = 0.002) variation in bacterial community, whereas the effect of host gender was insignificant (*P* = 0.103). The averaged AVD of bacterial communities in T1 (0.744 ± 0.016) was markedly increased (*P* < 0.05) compare with that in BP (0.752 ± 0.012). However, AVD values tended to be decreased over CRC progression (**Figure 2**).

### Distribution of core taxa

**Figure 3:**
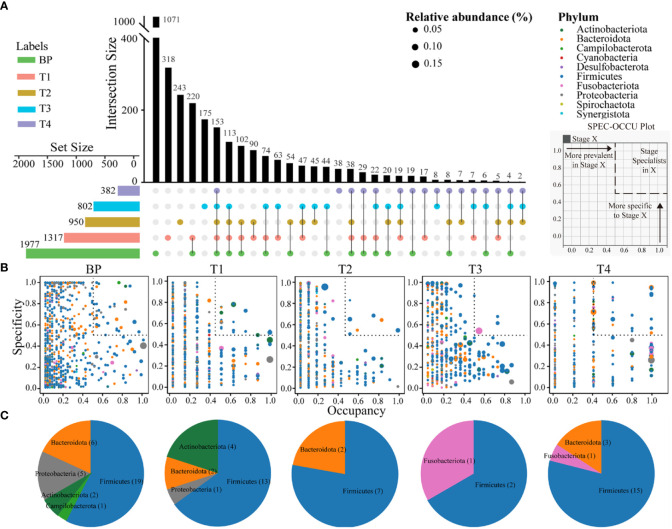
Upset plot displays the number of detected ASVs in each group (horizontal bars) and unique or shared ASVs (individual or connected points, respectively), in and among groups **(A)**. The SPEC-OCCU plots show 500 most abundant ASVs in each group; the x-axis represents occupancy, e.g., how well an ASV is distributed across biological replicates within each group; and the y-axis represents specificity, e.g., whether they are also found in other groups **(B)**. Pie charts showing the number of ASVs representing specialists in each group (See **Table S3** for the list of these specialists) **(C)**. Refer to **Figure 1** for abbreviations.

In total, 1071 ASVs were uniquely detected in BP. Intriguingly, the numbers of unique ASVs linearly decreased along CRC progression, with 318, 243, 175 and 38 ASVs in T1, T2, T3 and T4 tumors, respectively. Similarly, there were gradual decreases in shared ASVs between adjacent stages. For example, the groups with the highest number of shared ASVs were BP and T1 (220 ASVs), followed by 90 shared ASVs between T1 and T2, 45 shared ASVs between T2 and T3, with the least shared ASVs between T3 and T4 (8 ASVs) (**Figure 3A**). In addition, only 153 ASVs (accounting for 4.25% of all ASVs) were shared across the five groups, while 19 ASVs were consistently detected among the four CRC stages (**Figure 3A**). In addition, there was no significant difference in diversity among the five groups (**Figure S4**). These results indicated an increasing distinctness in bacterial communities over CRC progression.

To inspect how ASVs from each group are spread from BP to advanced CRC and also how specific they are to their stage, specificity and occupancy were calculated for each ASV, which were then projected onto a SPEC-OCCU biplot (**Figure 3B**). As indicated by the distribution of ASVs along the x-axis (occupancy), ASVs from BP displayed remarkably homogenous occupancy. To identify specialist taxa attributable to each group, we selected ASVs with specificity and occupancy higher or equal to 0.5 (dotted boxes in **Figure 3B**), that is, these ASVs are specific to a stage and common in their groups in most individuals. The number of these specialist ASVs substantially varied among groups. There was a decreasing trend in terms of observed richness from BP (33 ASVs represent), T1 (20 ASVs), T2 (9 ASVs) to T3 (3 ASVs) (**Table S3**), representing 1.7%, 1.5%, 0.95% and 0.37% of their total richness, respectively. Conversely, 19 specialist ASVs (representing 5.0%) were detected among the T4 tumors. Firmicutes species were the specialists across the all five groups (**Figure 3C**, **Table S3**).

### Identification of CRC driver taxa

**Figure 4:**
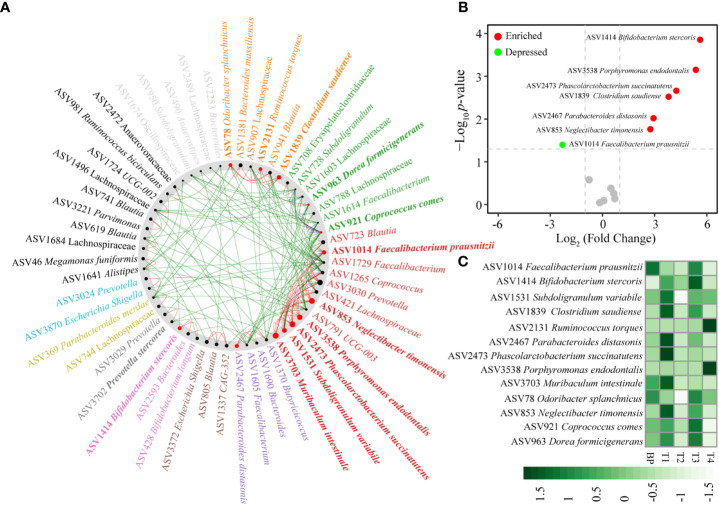
Identification of “driver” taxa from benign BP to CRC T1 tumor **(A)**, changes between T1 and BP **(B)**, distributions in BP and along the four CRC stages **(C)**. The nodes of the common sub-network were placed around a circle that was sorted by their identified community membership (in the CRC T1 network). All nodes belonging to the same community are assigned similar colors. Black nodes represent nodes that exist in both but interact directly with the common sub-network in either T1 tumor or BP. The size of the node is proportional to the NESH fraction of its scale, and the node is colored red if the intermediation of the node increases from BP to T1 tumor status. Thus, large red nodes are particularly important “driver” taxa. Refer to **Figure 1** for abbreviations.

Comparison of the gut networks between BP and CRC stage 1 (T1), an important step to tumorigenesis, 13 taxa drove the network shift from BP to initial CRC (**Figure 4A**, **Table S4**). Specifically, ASV2473 *Phascolarctobacterium succinatutens*, ASV3703 *Muribaculum intestinale*, ASV853 *Neglectibacter timonensis*, ASV3538 *Porphyromonas endodontalis* among others were the driver nodes (ASVs) with higher NESH scores (red color and bigger nodes) (**Figure 4A**, **Table S4**). Of the 13 driver taxa, the relative abundances of six taxa significantly enriched, and only ASV1014 *Faecalibacterium prausnitzii* depressed in T1 compared with BP (**Figure 4B**). In particular, the six enriched driver taxa were also the most abundant over CRC progression (**Figure 4C**). However, abundances of the remaining seven driver taxa insignificantly changed between BP and T1, such as ASV3538 *Porphyromonas endodontalis*, ASV2131 *Ruminococcus torques* and ASV78 *Odoribacter splanchnicus* (**Figure 4**, **Table S4**), while case studies have reported their enrichments in CRC patients (Flemer et al., 2017; Wolf et al., 2020). Based on the differential distribution across groups, 19 ASVs were spurted in CRC T1 tumors, which were substantially decreased in BP and along progressed CRC, including previously reported as CRC associated taxa, e.g., *Fusobacterium nucleatum* (**Figure S5A**). In addition, several potential pathogens, such as *Bacteroides fragilis*, *Clostridium perfringens* among others, were enriched and the most abundant in the advanced CRC stage 4 (T4) (**Figure S5B**).

### Establishment of diagnosis model for diagnosing CRC stages

We randomly selected 67 samples (training data, 32 BPs and 35 CRC patients) for constructing the diagnosis model. The remained 28 samples (test data, 13 BPs and 15 patients) were used for validation. In order to distinguish the CRC stage effect from the confounded roles of host age in governing the bacterial community, we firstly identified the top age-discriminatory lineages (**Figure S6**). In addition, to evaluate whether the diagnosis accuracy was influenced by taxonomic level, we screened the discriminatory lineages at the bacterial phylum, class, order, family, genus, or ASV level, respectively. To this end, we found that the CRC stage-discriminatory ASVs contributed the highest accuracy of classification after excluding host age effect (**Table 1**). For this reason, CRC stage-discriminatory ASVs were applied as dependent variables for diagnosing the BP and CRC stages in the final diagnosis model.

**Figure 5:**
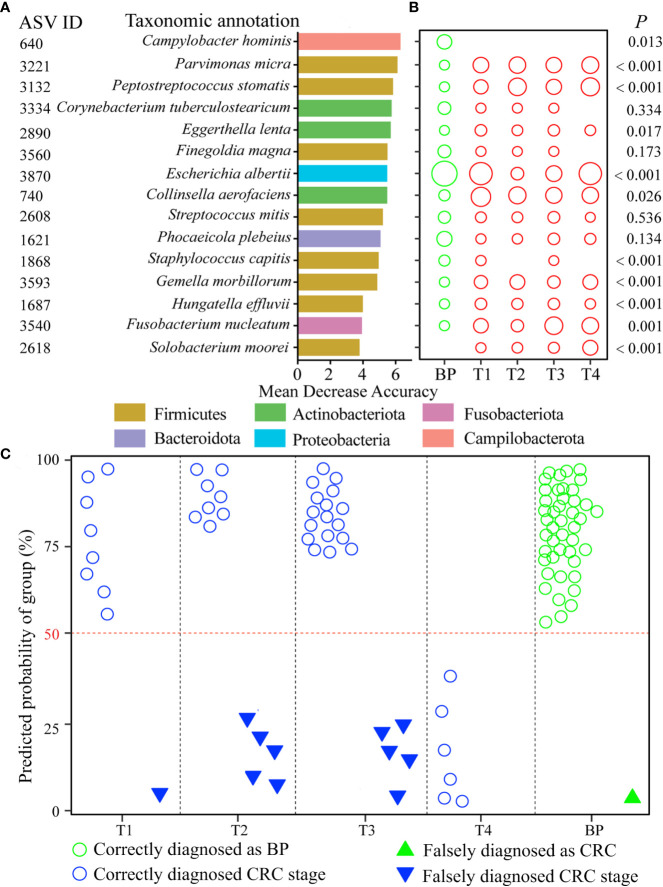
CRC diagnosis model using the CRC stage-discriminatory taxa. The top 15 CRC stage-discriminatory ASVs are ranked in descending order of importance to the accuracy of the diagnose model **(A)**. The diameters of the circles are proportional to the relative abundances of the 15 biomarkers **(B)**. The diagnosed CRC stages using profiles of the 15 CRC stage-discriminatory ASVs. The consistency between observed and diagnosed stage was termed a correct diagnosis with a cutoff of 50% **(C)**. Refer to **Figure 1** for abbreviations.

We screened nine age-discriminatory ASVs from the BP that contributed an overall 92.4% diagnosis accuracy (**Figure S6**). After excluding the nine age-discriminatory taxa, we identified 15 common CRC stage-discriminatory ASVs. To visualize these biomarkers, we constructed a phylogenetic tree to identify their closest species (**Figure S7**). For example, the most predictive taxon belonged to the *Campylobacter* genus based on decrease in “Mean Decrease Accuracy” coefficient (**Figure 5A**), which was phylogenetically affiliated with *Campylobacter hominis* with 99% similarity (**Figure S7**). In general, the relative abundances of the CRC stage-discriminatory ASVs were varied significantly (11 out of 15 ASVs) among the five groups (**Figure 5B**). Importantly, using the profiles of the 15 CRC stage-discriminatory ASVs as dependent variable, the diagnosis model contributed an overall 87.4% accuracy (**Table 1**). In BP, 44 samples (accounting for 97.8% of the controls) were correctly diagnosed as BP individuals. Among the CRC patients, 44 individuals (80.0% of patients) were predicted accurately as corresponding CRC stage (**Figure 5C**). Of note, no CRC patients were falsely diagnosed as BPs by using the 15 CRC stage-discriminatory ASVs, namely, no false-negative (**Table 1**). It is worthy to emphasize that the diagnosis model could accurately diagnose the initiation of CRC (T1 stage, 8 out of 9 cases, 88.9%). In addition, the falsely diagnosed samples were not related to host gender (**Figure S8A**), though the numbers of male patients were consistently higher than female individuals along the fours CRC stages (**Table S1**). Also, the diagnosis model were unbiased by host age (**Figure S8B**), while ages were markedly varied among patients. We also screened the CRC stage-discriminatory taxa without exclusion of the age-discriminatory taxa. We found that the model performance was unsatisfactory, with a marked decrease in diagnosis accuracy (71.6% vs. 87.4%, **Table S5**). In this regard, our optimization procedure was imperative and valuable, which substantially improved the accuracy of diagnosis model.

## Discussion

Currently there has been increased interest in the adenoma-specific markers that detect early CRC, partly due to the recognition that the bacterial communities are distinct along the control-adenoma-carcinoma sequence (Mizutani et al., 2020; Wu et al., 2021). By contrast, few studies have explored the bacterial communities over CRC progression, resulting in the uncertainty whether biomarkers could diagnose the four stages of CRC, rather than case-control. Additionally, as microbes are implicated in colorectal carcinogenesis, development and treatment outcome (Jin et al., 2019; Montalban-Arques and Scharl, 2019; Mizutani et al., 2020; Ting et al., 2022), understanding the dynamics in CRC development is a necessary initial step to developing a more complete understanding of both the ecology and etiology.

Increasingly evidence has shown that faecal bacterial communities are distinguishable from individuals with CRC or adenomas to controls (Wang et al., 2012; Flemer et al., 2018; Wu et al., 2021). In contrast, there is still lack of data about the bacterial profiles over CRC progression, especially these at cancerous tissue. Considering the functional importance of microbes in promoting CRC tumorigenesis (Cheng et al., 2020; Mizutani et al., 2020), and given that faecal microbiota only partially reflects mucosal counterpart in CRC (Flemer et al., 2017), this ignorance could bias the identification of CRC associated taxa. Trying to comprehensively collect the tumors comprising the four CRC stages, our results depicted that there were distinct segregations in bacterial community structure between polyps and tumors, and along the four CRC stages (**Figure 1**, **Table S2**). These patterns suggest that, despite the extensive physical variances among individuals (e.g., age, gender, and CEA level, **Table S1** and **Figure S1**), each stage exerts sufficiently unique conditions to assemble communities that are consistent in structure according to BP or CRC stage.

As the stability of the microbiota could be affected by host disease (Xiong, 2018; Aho et al., 2019), we compared with the stability of bacterial communities over CRC progression. CRC initiation (T1 stage) sharply disrupted the stability of bacterial community compared with BPs (**Figure 2**). There are several possible explanations for this pattern. First, the bloom of pathogenic taxa outcompete resident commensals, as supported by increased abundances of known pathogens in T1 tumors, such as *Fusobacterium nucleatum*, *Bacteroides fragilis*, and *Escherichia fergusonii* (**Figure S6**). Second, pathogen invasion could attenuate host filtering on the colonization of external taxa, leading to a dominance of stochastic processes (e.g., random birth and death) governing community assembly (Mallon et al., 2015; Adair and Douglas, 2017). Accordingly, there was a heterogeneous bacterial community (higher AVD value) in T1 tumors (**Figure 2**). However, as disease severity increased, inflammatory microenvironment could exert the dominant role of homogeneous selection, resulting in a convergent, disease-like microbial community (Subramanian et al., 2014; Xiong et al., 2017). Consistent with this assertion, the AVD values linearly decreased along CRC progression (**Figure 2**), whereas phylogenetic diversity exhibited an opposing trend (**Figure S4B**). Given that microbes are implicated in the outcome of CRC therapy (Montalban-Arques and Scharl, 2019; Mizutani et al., 2020; Ting et al., 2022), the convergence in tumor bacterial community may partially explain why advanced CRC is difficult to be curative.

It has proposed a stratification of individuals into three distinct enterotypes (Arumugam et al., 2011), while others supported a concept of stratification based on bacterial abundance gradients (Flemer et al., 2017). We detected a stratification of individuals between distinct enterotypes and abundance gradients, as supported by distinct structures of bacterial communities when integrated the abundance of ASVs, e.g., abundance gradients (**Figure 1**, **Table S2**). However, we also found rapid replacement of ASVs over CRC progression, because there were linearly deceased numbers and low proportions of overlapped ASVs between BP and advanced CRC, as well as shuffling between adjacent two stages, e.g., distinct enterotypes (**Figure 3A**). This is apparent in the SPEC-OCCU plots (**Figure 3B**), where the majority of the ASVs exhibited low occupancy, indicating that few of them are consistently detected among individuals. In accordance, the tumor communities harbored significantly lower homogeneity among individuals in CRC T1, T2 and T3 stages, compared with these in BP (**Figure 2**). These findings indicate that the tumor environments are less selective for a stable core community, resulting in heterogeneity in bacterial communities among patients. Considering that microbes determine the therapeutic efficiency of CRC, the heterogeneity in bacterial communities may guide the design of personalized medicine (Kong et al., 2019; Shi et al., 2020; Ting et al., 2022). Although this study was not designed to evaluate treatment outcome, clearly, there is a pressing need for longitudinal study exploring the associations between the tumor microbiota and treatment response in CRC patients.

Given that the gut microbiome is an important etiological element in the initiation and progression of CRC (Montalban-Arques and Scharl, 2019; Cheng et al., 2020; Mizutani et al., 2020; Guo et al., 2022), sufficient and accurate identification of CRC associated taxa could facilitate the targets for diagnosis and therapy. We identified the well known promoters in colorectal carcinogenesis, *Fusobacterium nucleatum* (Rubinstein et al., 2013), based on its sharply increased abundance in T1 stage tumors (**Figure 5A**). Similarly, three well known CRC-associated taxa, *F. nucleatum*, *Bacteroides fragilis*, and *Campylobacter concisus* (Guo et al., 2022), were enriched in the advanced T4 stage tumors (**Figure 5B**). Thus, there is a lack of consistency in the bacterial taxa associated with CRC progression, in accordance with studies conducted previously (Feng et al., 2015; Mizutani et al., 2020). This pattern supports the so-called “driver-passenger” model, which proposes that different bacteria sequentially implicate in CRC tumor initiation and progression. The “driver” bacteria are replaced by “passenger” bacteria that are better adapted to the conditions in and around cancerous cells (Tjalsma et al., 2012; Tilg et al., 2018). Of note, other bloomed ASVs have not been reported to be associated with CRC, instead, a few ASVs could be potential probiotics, such as *Corynebacterium vitaeruminis* (Colombo et al., 2017), and *Streptococcus alactolyticus* (Zhang et al., 2021). In this regard, it is cautious to identify CRC-associated taxa by increased abundance in the tumors. Going forward, we identified 13 “driver” bacterial ASVs by the NetShift model, moving beyond differential abundance, of which 6 ASVs were significantly enriched and the most abundant in T1 tumors (**Figure 4**). Among the 6 ASVs, ASV1839 *Clostridium saudiense* is recently identified as an opportunistic pathogen with the potential to cause hepatocellular carcinoma, which translocates from the gastrointestinal system to biliary system (Yoon et al., 2022). Unexpectedly, ASV1414 *Bifidobacterium stercoris* and ASV2467 *Parabacteroides distasonis* were significantly enriched in T1 tumors (**Figure 4B**). *B. stercoris* is able to produce acetic acid and lactic acid, and promote antitumor immunity (Sivan et al., 2015), while the later could attenuate toll-like receptor 4 signaling and thus blocks colon tumor formation (Koh et al., 2018). One possible explanation for this counterintuitive pattern is that the hosts release specific chemicals that favor the recruitment of beneficial microbes or of antagonists able to suppress the growth of pathogens, according to the “cry-for-help” hypothesis (Rolfe et al., 2019), although further study is needed to validate its applicability in human diseases. Conversely, the butyrate-producing AVS1014 *Faecalibacterium prausnitzii* is an anti-inflammatory commensal bacterium (Miquel et al., 2013), thus its reduction is expected to be associated with CRC. Consistent with the above, the ratio of *Fusobacterium nucleatum* to *Faecalibacterium prausnitzii* has been identified as a valuable biomarker for screening early CRC (Guo et al., 2018). The beneficial ASVs decreased rapidly along advanced CRC (**Figures 4**, **S5**), which raises the intriguing possibility that tumors could recruit beneficial taxa to antagonize CRC-associated pathogens at the initial stage. Collectively, the taxa that trigger protumorigenic environments could be attributed to the enrichment of pathogenic strains, and also the depletion of probiotic species.

The establishment of gut microbiota—host health relationship and modeling algorithms facilitates the identification of bio-indicators diagnosing disease severity, which is a key goal of microbiome research. Multiple studies have extensively shown human age to be a strong covariate of the gut microbiota (Falony et al., 2016; Greenhalgh et al., 2016), thus host age may overshadow changes in commensals associated with CRC. For this reason, we teased apart the effect of ageing-related and CRC-related changes in the bacterial community. To achieve this, we identified age-discriminatory ASVs that featured the age of BP controls. The top nine age-discriminatory ASVs contributed a high consistency (*r* = 0.924, *P* < 0.001) between diagnosed and chronologic ages (**Figure S6**). After removal of the nine age-discriminatory ASVs, we further identified CRC stage-discriminatory taxa for diagnosing BPs and the four CRC stages, with an overall 87.4% accuracy. It is worthy to emphasize that this optimization substantially improves the performance of our diagnosis model compared with the model neglecting age effect (71.6% accuracy, **Table S5**). As a result, the diagnosis accuracy was not affected by host age (**Figure S8**). Our diagnosis model could add the designation of CRC stage-dependent clinical trials, especially the patients with poor pathological feature and un-reproducibility between two doctors (Quirke et al., 2007). Similarly and more importantly, no CRC patients were falsely diagnosed as BPs (**Table 1**), illustrating that there was no false-negative in diagnosing CRC when ignored the CRC stage. In this regard, our diagnosis model is reliable to warn the cancerization of CRC with 100% accuracy, which in turn alerts patients to further treatment. One might argue that the low sampling sizes at the T1 (*N* = 9) and T4 (*N* = 6) stages (**Table S1**), which could weaken statistics power. Here we collected unique and comprehensive tumors (four CRC stages), instead of fecal samples. We want to point out the CRC tend to be inspected at later stages, while T4 patients generally refuse surgery. Consequently, we collected limited tumors from T1 and T4 patients. However, the highly diagnosed accuracy of patients at the two stages indicated that our diagnosis model was not strongly affected by the sampling sizes (**Table 1**). Despite high heterogeneity of bacterial communities among patients at each CRC stage (**Figure 1**), we are surprised that, given the high number of ASVs (*N* = 3,601) identified across the 95 individuals, only 15 ASVs (accounting for 0.4%) accurately discriminated human BPs and CRC stages. This small subset affords unique experimental opportunities that could be prioritized based on their feature importances to the diagnosis model (**Figure 5**). For example, it has been shown that levels of *Parvimonas micra* in faecal samples from CRC patients are significantly higher compared to healthy individuals (Löwenmark et al., 2020), as also observed in the present study (**Figures 5A, B**). Thus, *P*. *micra* is proposed as non-invasive biomarkers for CRC (Löwenmark et al., 2020). Similarly, a metagenomic analysis of faecal microbiome reveals that *P*. *micra* and *Solobacterium moreii* are positively associated with CRC (Yu et al., 2017). Besides confirming known associations of ASV3221 *P*. *micra*, ASV2618 *S. moreii*, ASV3540 *Fusobacterium nucleatum* and ASV3132 *Peptostreptococcus stomatis* with CRC, we found significant associations with several species, including ASV640 *Campylobacter hominis*, ASV740 *Collinsella aerofaciens* among others (**Figure 5**). Clearly, the pressing steps would be to isolate representatives of these CRC stage-discriminatory ASVs and to explore their roles in CRC carcinogenesis.

## Conclusion

This is the few attempts to explore CRC-associated taxa and underlying mechanisms in CRC progression from an ecological perspective. Tumor microenvironments are less selective for stable core community, leading to heterogeneity in bacterial communities among patients, as supported by higher AVD, lower occupancy and specificity. However, tumors could recruit beneficial taxa to antagonize CRC-associated pathogens at the initial stage, in accordance with the “cry-for-help” pattern that has not been recognized before. Based on the “driver-passenger” distribution of CRC-associated ASVs, CRC initiation could be attributed to the enrichment of pathogenic strains, and also the depletion of probiotic species. By distinguishing age- from CRC stage-associated taxa, the diagnosis model for accurately diagnoses (an overall 87.4% accuracy) BPs and the four CRC stages, especially without false-negative for CRC patients. The diagnosis model could add CRC stage stratification, especially the patients with poor pathological feature and un-reproducibility between doctors. Additional works will be imperative to eventually validate them in much larger cohorts before clinical deployment.

## Data availability statement

The datasets presented in this study can be found in online repositories. The names of the repository/repositories and accession number(s) can be found in the article/**Supplementary Material**.

## Ethics statement

The studies involving human participants were reviewed and approved by the ethics committees of Hwa Mei Hospital, University of Chinese Academy of Science (No. PJ-NBEY-KY-2020-042-01). The patients/participants provided their written informed consent to participate in this study.

## Author contributions

XD, JL and JX conceived and designed the research. PC, JL, and HS conducted the experiments. JX contributed analytical tools. PC, JL, and HS analyzed the data. PC wrote the manuscript with help from JX, JL and XD. All authors contributed to the article and approved the submitted version.

## References

[B1] Adair K. L. Douglas A. E. (2017). Making a microbiome: The many determinants of host-associated microbial community composition. Curr. Opin. Microbiol. 35, 23–29. doi: 10.1016/j.mib.2016.11.002 27907842

[B2] Aho V. T. Pereira P. A. Voutilainen S. Paulin L. Pekkonen E. Auvinen P.. (2019). Gut microbiota in parkinson's disease: Temporal stability and relations to disease progression. EBioMedicine 44, 691–707. doi: 10.1016/j.ebiom.2019.05.064 31221587 PMC6606744

[B3] Anderson M. J. Willis T. J. (2003). Canonical analysis of principal coordinates: a useful method of constrained ordination for ecology. Ecology 84, 511–525. doi: 10.1890/0012-9658(2003)084[0511:CAOPCA]2.0.CO;2

[B4] Arnold M. Sierra M. S. Laversanne M. Soerjomataram I. Jemal A. Bray F. (2017). Global patterns and trends in colorectal cancer incidence and mortality. Gut 66, 683–691. doi: 10.1136/gutjnl-2015-310912 26818619

[B5] Arumugam M. Raes J. Pelletier E. Le Paslier D. Yamada T. Mende D. R.. (2011). Enterotypes of the human gut microbiome. Nature 473, 174–180. doi: 10.1038/nature09944 21508958 PMC3728647

[B6] Bolyen E. Rideout J. R. Dillon M. R. Bokulich N. A. Abnet C. C. Al-Ghalith G. A.. (2019). Reproducible, interactive, scalable and extensible microbiome data science using QIIME 2. Nat. Biotechnol. 37, 852–857. doi: 10.1038/s41587-019-0209-9 31341288 PMC7015180

[B7] Bray F. Ferlay J. Soerjomataram I. Siegel R. Torre L. Jemal A. (2018). GLOBOCAN estimates of incidence and mortality worldwide for 36 cancers in 185 countries. CA Cancer J. Clin. 68, 394–424. doi: 10.3322/caac.21492 30207593

[B8] Bridges K. M. Greiner K. A. Umar S. (2019). Deciphering the colorectal cancer gut microbiota: association vs. causality. Curr. Col. Cancer Rep. 15, 70–77. doi: 10.1007/s11888-019-00431-5

[B9] Cai Z. Liu Q. (2021). Understanding the global cancer statistics 2018: implications for cancer control. Sci. China Life Sci. 64, 1017–1020. doi: 10.1007/s11427-019-9816-1 31463738

[B10] Callahan B. J. McMurdie P. J. Rosen M. J. Han A. W. Johnson A. A. Holmes S. P. (2016). DADA2: High-resolution sample inference from illumina amplicon data. Nat. Methods 13, 581–583. doi: 10.1038/nmeth.3869 27214047 PMC4927377

[B11] Cheng Y. Ling Z. Li L. (2020). The intestinal microbiota and colorectal cancer. Front. Immunol. 11, 615056. doi: 10.3389/fimmu.2020.615056 33329610 PMC7734048

[B12] Coker O. O. Liu C. Wu W. K. Wong S. H. Jia W. Sung J. J.. (2022). Altered gut metabolites and microbiota interactions are implicated in colorectal carcinogenesis and can be non-invasive diagnostic biomarkers. Microbiome 10, 35. doi: 10.1186/s40168-021-01208-5 35189961 PMC8862353

[B13] Colombo M. Castilho N. Todorov S. Nero L. (2017). Beneficial and safety properties of a corynebacterium vitaeruminis strain isolated from the cow rumen. Probiotics Antimicr. 9, 157–162. doi: 10.1007/s12602-017-9263-0 28258546

[B14] Dai W. Chen J. Xiong J. (2018). Concept of microbial gatekeepers positive guys. Appl. Microbiol. Biotechnol. 103, 633–641. doi: 10.1007/s00253-018-9522-3 30465305

[B15] Dufrêne M. Legendre P. (1997). Species assemblages and indicator species: the need for a flexible asymmetrical approach. Ecol. Monogr. 67, 345–366. doi: 10.2307/2963459

[B16] Falony G. Joossens M. Vieira-Silva S. Wang J. Darzi Y. Faust K.. (2016). Population-level analysis of gut microbiome variation. Science 352, 560–564. doi: 10.1126/science.aad3503 27126039

[B17] Feng Q. Liang S. Jia H. Stadlmayr A. Tang L. Lan Z.. (2015). Gut microbiome development along the colorectal adenoma-carcinoma sequence. Nat. Commun. 6, 6528. doi: 10.1038/ncomms7528 25758642

[B18] Flanagan L. Schmid J. Ebert M. Soucek P. Kunicka T. Liska V.. (2014). *Fusobacterium nucleatum* associates with stages of colorectal neoplasia development, colorectal cancer and disease outcome. Eur. J. Cin. Microbiol. Infect. Dis. 33, 1381–1390. doi: 10.1007/s10096-014-2081-3 24599709

[B19] Flemer B. Lynch D. B. Brown J. M. Jeffery I. B. Ryan F. J. Claesson M. J.. (2017). Tumour-associated and non-tumour-associated microbiota in colorectal cancer. Gut 66, 633–643. doi: 10.1136/gutjnl-2015-309595 26992426 PMC5529966

[B20] Flemer B. Warren R. D. Barrett M. P. Cisek K. Das A. Jeffery I. B.. (2018). The oral microbiota in colorectal cancer is distinctive and predictive. Gut 67, 1454–1463. doi: 10.1136/gutjnl-2017-314814 28988196 PMC6204958

[B21] Fong W. Li Q. Yu J. (2020). Gut microbiota modulation: A novel strategy for prevention and treatment of colorectal cancer. Oncogene 39, 4925–4943. doi: 10.1038/s41388-020-1341-1 32514151 PMC7314664

[B22] Fushiki T. (2011). Estimation of prediction error by using K-fold cross-validation. Stat. Comput. 21, 137–146. doi: 10.1007/s11222-009-9153-8

[B23] Ghosh T. S. Das M. Jeffery I. B. O'Toole P. W. (2020). Adjusting for age improves identification of gut microbiome alterations in multiple diseases. eLife 9, e50240. doi: 10.7554/eLife.50240 32159510 PMC7065848

[B24] Greenhalgh K. Meyer K. M. Aagaard K. M. Wilmes P. (2016). The human gut microbiome in health: establishment and resilience of microbiota over a lifetime. Environ. Microbiol. 18, 2103–2116. doi: 10.1111/1462-2920.13318 27059297 PMC7387106

[B25] Guo S. Li L. Xu B. Li M. Zeng Q. Xiao H.. (2018). A simple and novel fecal biomarker for colorectal cancer: ratio of fusobacterium nucleatum to probiotics populations, based on their antagonistic effect. Clin. Chem. 64, 1327–1337. doi: 10.1373/clinchem.2018.289728 29914865

[B26] Guo Q. Qin H. Liu X. Zhang X. Chen Z. Qin T.. (2022). The emerging roles of human gut microbiota in gastrointestinal cancer. Front. Immunol. 13, 915047. doi: 10.3389/fimmu.2022.915047 35784372 PMC9240199

[B27] Gweon H. S. Bowes M. J. Moorhouse H. L. Oliver A. E. Bailey M. J. Acreman M. C.. (2021). Contrasting community assembly processes structure lotic bacteria metacommunities along the river continuum. *Environ* . Microbiol 23, 484–498. doi: 10.1111/1462-2920.15337 PMC7898806 33258525

[B28] Koh G. Y. Kane A. Lee K. Xu Q. Wu X. Roper J.. (2018). Parabacteroides distasonis attenuates toll-like receptor 4 signaling and akt activation and blocks colon tumor formation in high-fat diet-fed azoxymethane-treated mice. Int. J. Cancer 143, 1797–1805. doi: 10.1002/ijc.31559 29696632

[B29] Kong C. Gao R. Yan X. Huang L. He J. Li H.. (2019). Alterations in intestinal microbiota of colorectal cancer patients receiving radical surgery combined with adjuvant CapeOx therapy. Sci. China Life Sci. 62, 1178–1193. doi: 10.1007/s11427-018-9456-x 30796721

[B30] Kuntal B. K. Chandrakar P. Sadhu S. Mande S. S. (2019). 'NetShift': a methodology for understanding 'driver microbes' from healthy and disease microbiome datasets. ISME J. 13, 442–454. doi: 10.1038/s41396-018-0291-x 30287886 PMC6331612

[B31] Lex A. Gehlenborg N. Strobelt H. Vuillemot R. Pfister H. (2014). UpSet: visualization of intersecting sets. IEEE Trans. Vis. Comput. Graph 20, 1983–1992. doi: 10.1109/TVCG.2014.2346248 26356912 PMC4720993

[B32] Li J. Zhang A. Wu F. Wang X. (2022). Alterations in the gut microbiota and their metabolites in colorectal cancer: Recent progress and future prospects. Front. Oncol. 12, 841552. doi: 10.3389/fonc.2022.841552 35223525 PMC8875205

[B33] Liaw A. Wiener M. (2002). Classification and regression by randomForest. R News 2, 18–22.

[B34] Löwenmark T. Löfgren-Burström A. Zingmark C. Eklöf V. Dahlberg M. Wai S. N.. (2020). *Parvimonas micra* as a putative non-invasive faecal biomarker for colorectal cancer. Sci. Rep. 10, 15250. doi: 10.1038/s41598-020-72132-1 32943695 PMC7499209

[B35] Mallon C. A. Van Elsas J. D. Salles J. F. (2015). Microbial invasions: the process, patterns, and mechanisms. Trends Microbiol. 23, 719–729. doi: 10.1016/j.tim.2015.07.013 26439296

[B36] Marchesi J. R. Adams D. H. Fava F. Hermes G. D. Hirschfield G. M. Hold G.. (2016). The gut microbiota and host health: A new clinical frontier. Gut 65, 330–339. doi: 10.1136/gutjnl-2015-309990 26338727 PMC4752653

[B37] Miquel S. Martin R. Rossi O. Bermúdez-Humarán L. Chatel J. Sokol H.. (2013). Faecalibacterium prausnitzii and human intestinal health. Curr. Opin. Microbiol. 16, 255–261. doi: 10.1016/j.mib.2013.06.003 23831042

[B38] Mizutani S. Yamada T. Yachida S. (2020). Significance of the gut microbiome in multistep colorectal carcinogenesis. Cancer Sci. 111, 766–773. doi: 10.1111/cas.14298 31910311 PMC7060472

[B39] Montalban-Arques A. Scharl M. (2019). Intestinal microbiota and colorectal carcinoma: Implications for pathogenesis, diagnosis, and therapy. EBioMedicine 48, 648–655. doi: 10.1016/j.ebiom.2019.09.050 31631043 PMC6838386

[B40] Oksanen J. Blanchet F. G. Friendly M. Kindt R. Legendre P. McGlinnLan D.. (2018). Vegan: community ecology package. R package version, 2, 5–3. Available at: https://CRAN.Rproject.org/package=vegan

[B41] Quast C. Pruesse E. Yilmaz P. Gerken J. Schweer T. Yarza P.. (2012). The SILVA ribosomal RNA gene database project: improved data processing and web-based tools. Nucleic Acids Res. 41, D590–D596. doi: 10.1093/nar/gks1219 23193283 PMC3531112

[B42] Quirke P. Williams G. T. Ectors N. Ensari A. Piard F. Nagtegaal I. (2007). The future of the TNM staging system in colorectal cancer: Time for a debate? Lancet Oncol. 8, 651–657. 17613427 10.1016/S1470-2045(07)70205-X

[B43] Rolfe S. A. Griffiths J. Ton J. (2019). Crying out for help with root exudates: adaptive mechanisms by which stressed plants assemble health-promoting soil microbiomes. Curr. Opin. Microbiol. 49, 73–82. doi: 10.1016/j.mib.2019.10.003 31731229

[B44] Rubinstein M. R. Wang X. Liu W. Hao Y. Cai G. Han Y. W. (2013). Fusobacterium nucleatum promotes colorectal carcinogenesis by modulating e-cadherin/β-catenin signaling *via* its FadA adhesin. Cell Host Microbe 14, 195–206. doi: 10.1016/j.chom.2013.07.012 23954158 PMC3770529

[B45] Shah M. S. DeSantis T. Z. Weinmaier T. McMurdie P. J. Cope J. L. Altrichter A.. (2018). Leveraging sequence-based faecal microbial community survey data to identify a composite biomarker for colorectal cancer. Gut 67, 882–891. doi: 10.1136/gutjnl-2016-313189 28341746

[B46] Shi W. Shen L. Zou W. Wang J. Yang J. Wang Y.. (2020). The gut microbiome is associated with therapeutic responses and toxicities of neoadjuvant chemoradiotherapy in rectal cancer patients–a pilot study. Front. Cell. Infect. Microbiol. 10, 562463. doi: 10.3389/fcimb.2020.562463 33363048 PMC7756020

[B47] Sivan A. Corrales L. Hubert N. Williams J. B. Aquino-Michaels K. Earley Z. M.. (2015). Commensal bifidobacterium promotes antitumor immunity and facilitates anti–PD-L1 efficacy. Science 350, 1084–1089. doi: 10.1126/science.aac4255 26541606 PMC4873287

[B48] Subramanian S. Huq S. Yatsunenko T. Haque R. Mahfuz M. Alam M. A.. (2014). Persistent gut microbiota immaturity in malnourished Bangladeshi children. Nature 510, 417–421. doi: 10.1038/nature13421 24896187 PMC4189846

[B49] Takahashi S. Tomita J. Nishioka K. Hisada T. Nishijima M. (2014). Development of a prokaryotic universal primer for simultaneous analysis of bacteria and archaea using next-generation sequencing. PloS One 9, e105592. doi: 10.1371/journal.pone.0105592 25144201 PMC4140814

[B50] Tilg H. Adolph T. E. Gerner R. R. Moschen A. R. (2018). The intestinal microbiota in colorectal cancer. Cancer Cell 33, 954–964. doi: 10.1016/j.ccell.2018.03.004 29657127

[B51] Ting N. L. Lau H. C. Yu J. (2022). Cancer pharmacomicrobiomics: targeting microbiota to optimise cancer therapy outcomes. Gut 71, 1412–1425. doi: 10.1136/gutjnl-2021-326264 35277453 PMC9185832

[B52] Tjalsma H. Boleij A. Marchesi J. R. Dutilh B. E. (2012). A bacterial driver–passenger model for colorectal cancer: beyond the usual suspects. Nat. Rev. Microbiol. 10, 575–582. doi: 10.1038/nrmicro2819 22728587

[B53] Wang T. Cai G. Qiu Y. Fei N. Zhang M. Pang X.. (2012). Structural segregation of gut microbiota between colorectal cancer patients and healthy volunteers. ISME J. 6, 320–329. doi: 10.1038/ismej.2011.109 21850056 PMC3260502

[B54] Wolf P. G. Kolosslov V. Zhou Z. Ly L. Doden H. Devendran S.. (2020). The colorectal cancer associated microbe odoribacter splanchnicus produces genotoxic hydrogen sulfide *via* cysteine metabolism. Cancer Res. 80, 3342–3342. doi: 10.1158/1538-7445.AM2020-3342

[B55] Wu Y. Jiao N. Zhu R. Zhang Y. Wu D. Wang A.-J.. (2021). Identification of microbial markers across populations in early detection of colorectal cancer. Nat. Commun. 12, 3063. doi: 10.1038/s41467-021-23265-y 34031391 PMC8144394

[B56] Xie Y. H. Gao Q. Y. Cai G. X. Sun X. M. Zou T. H. Chen H. M.. (2017). Fecal *Clostridium symbiosum* for noninvasive detection of early and advanced colorectal cancer: test and validation studies. EBioMedicine 25, 32–40. doi: 10.1016/j.ebiom.2017.10.005 29033369 PMC5704049

[B57] Xiong J. (2018). Progress in the gut microbiota in exploring shrimp disease pathogenesis and incidence. Appl. Microbiol. Biotechnol. 102, 7343–7350. doi: 10.1007/s00253-018-9199-7 29982924

[B58] Xiong J. Zhu J. Dai W. Dong C. Qiu Q. Li C. (2017). Integrating gut microbiota immaturity and disease-discriminatory taxa to diagnose the initiation and severity of shrimp disease. Environ. Microbiol. 19, 1490–1501. doi: 10.1111/1462-2920.13701 28205371

[B59] Xun W. Liu Y. Li W. Ren Y. Xiong W. Xu Z.. (2021). Specialized metabolic functions of keystone taxa sustain soil microbiome stability. Microbiome 9, 1–15. doi: 10.1186/s40168-020-00985-9 33517892 PMC7849160

[B60] Yoon E. Kim T. Y. Heo W. Y. Kang O. Yu H. Lee J. H.. (2022). The first case of *Clostridium saudiense* bacteremia in a patient with hepatocellular carcinoma. Ann. Lab. Med. 42, 491–493. doi: 10.3343/alm.2022.42.4.491 35177573 PMC8859549

[B61] Yu J. Feng Q. Wong S. H. Zhang D. yi Liang Q. Qin Y.. (2017). Metagenomic analysis of faecal microbiome as a tool towards targeted non-invasive biomarkers for colorectal cancer. Gut 66, 70–78. doi: 10.1136/gutjnl-2015-309800 26408641

[B62] Zhang J. Zhang H. Wang L. Zhang K. Qiu Z. Zhang K.. (2021). The safety and potential probiotic properties analysis of *Streptococcus alactolyticu*s strain FGM isolated from the chicken cecum. Ann. Microbiol. 71, 19. doi: 10.1186/s13213-021-01630-y

