# Supplementary material for: Tumor bacterial markers diagnose the initiation and four stages of colorectal cancer
Source: Front Cell Infect Microbiol. 2023 Mar 13;13:1123544. doi: 10.3389/fcimb.2023.1123544 (PMC10040638; doi:10.3389/fcimb.2023.1123544)

**Supplemental data summary**

**Tumor Bacterial Markers Diagnose the Initiation and Four** **Stages of Colorectal Cancer**

*Ping Cai^1,2,^, Jinbo Xiong ^3,4^, Haonan Sha ^3,4^, Xiaoyu Dai^1,2*^ and Jiaqi Lu ^3,4*^*

*^1^ Ningbo Second Hospital, Ningbo 315000, China*

*^2^ NingboInstitute of Life and Health Industry, University of Chinese Academy of Sciences, Ningbo 315000, China*

*^3^* *State Key Laboratory for Managing Biotic and Chemical Threats to the Quality and Safety of Agro-products, Ningbo University, Ningbo 315211, China*

*^4^ Key Laboratory of Marine Biotechnology of Zhejiang Province, School of Marine Sciences, Ningbo University, Ningbo 315211, China*

***Corresponding authors**

Xiaoyu Dai, E-mail address: daixiaoyu1968@163.com

Jiaqi Lu, E-mail address: 17855822589@163.com

**Table S1** The clinicopathological classification of 50 colorectal cancer (CRC) patients and 45 benign polyps.

| Stage | Male | Female | Total |
| --- | --- | --- | --- |
| Benign polyps (BP) | 26 | 19 | 45 |
| T1 | 9 | 0 | 9 |
| T2 | 11 | 2 | 13 |
| T3 | 18 | 4 | 22 |
| T4 | 6 | 0 | 6 |
| Total | 70 | 25 | 95 |

**Note:** Colorectal cancer (CRC) stages were categorized into T1, T2, T3 and T4 based on Tumour-nodes-metastasis (TNM) staging system.

**Table S2** Community dissimilarity between each paired gut bacterial communities based on non-parametric multivariate analysis of variance (NPMANOVA) using Bray-Curtis distance. Top diagonal cells are *F* values, and lower diagonal cells are *P* values. Refer to Table S1 for abbreviations.

| CRC stage | T1 | T2 | T3 | T4 | BP |
| --- | --- | --- | --- | --- | --- |
| T1 |  | 2.16 | 1.45 | 2.51 | 3.05 |
| T2 | 0.0044 |  | 1.50 | 2.35 | 5.85 |
| T3 | 0.0713 | 0.0476 |  | 2.38 | 5.43 |
| T4 | 0.0043 | 0.0100 | 0.0005 |  | 4.33 |
| BP | 0.0003 | 0.0001 | 0.0001 | 0.0001 |  |

| **Table S3** List of the specialists and their relative abundances in benign polyps (BP) and over the four CRC stages, T1, T2, T3 and T4 | | | | | |  |  |
| --- | --- | --- | --- | --- | --- | --- | --- |
| ASV ID and Taxonomy | BP | T1 | T2 | T3 | T4 |  |  |
| ASV1729 Firmicutes; Clostridia; Oscillospirales; Ruminococcaceae; Faecalibacterium | 3.183 |  |  |  |  |  |  |
| ASV1690 Bacteroidota; Bacteroidia; Bacteroidales; Bacteroidaceae; Bacteroides | 2.058 |  |  |  |  |  |  |
| ASV1621 Bacteroidota; Bacteroidia; Bacteroidales; Bacteroidaceae; Bacteroides; Bacteroideplebeius | 1.923 |  |  |  |  |  |  |
| ASV640 Campilobacterota; Campylobacteria; Campylobacterales; Campylobacteraceae; Campylobacter hominis | 1.572 |  |  |  |  |  |  |
| ASV3683 Bacteroidota; Bacteroidia; Bacteroidales; Prevotellaceae; Prevotella | 1.364 |  |  |  |  |  |  |
| ASV2593 Bacteroidota; Bacteroidia; Bacteroidales; Bacteroidaceae; Bacteroides | 1.178 |  |  |  |  |  |  |
| ASV1700 Firmicutes; Clostridia; Lachnospirales; Lachnospiraceae; Dorea | 0.924 |  |  |  |  |  |  |
| ASV164 Bacteroidota; Bacteroidia; Bacteroidales; Bacteroidaceae; Bacteroides; Bacteroidecoprocola | 0.734 |  |  |  |  |  |  |
| ASV3560 Firmicutes; Clostridia; Peptostreptococcales-Tissierellales; Finegoldia; uncultured_bacterium | 0.692 |  |  |  |  |  |  |
| ASV3163 Proteobacteria; Gammaproteobacteria; Pasteurellales; Pasteurellaceae; Haemophilus | 0.644 |  |  |  |  |  |  |
| ASV3334 Actinobacteriota; Actinobacteria; Corynebacteriales; Corynebacteriaceae; Corynebacterium | 0.628 |  |  |  |  |  |  |
| ASV3869 Proteobacteria; Gammaproteobacteria; Enterobacterales; Morganellaceae; Proteus | 0.549 |  |  |  |  |  |  |
| ASV1397 Firmicutes; Clostridia; Peptostreptococcales-Tissierellales; Peptostreptococcaceae; Intestinibacter | 0.499 |  |  |  |  |  |  |
| ASV1270 Bacteroidota; Bacteroidia; Bacteroidales; Bacteroidaceae; Bacteroides; Bacteroidemassiliensis | 0.471 |  |  |  |  |  |  |
| ASV3576 Firmicutes; Clostridia; Peptostreptococcales-Tissierellales; Fenollaria; uncultured_bacterium | 0.433 |  |  |  |  |  |  |
| ASV3554 Firmicutes; Clostridia; Peptostreptococcales-Tissierellales; Peptoniphilus | 0.423 |  |  |  |  |  |  |
| ASV2001 Firmicutes; Clostridia; Peptostreptococcales-Tissierellales; Anaerococcus; Anaerococcuvaginalis | 0.393 |  |  |  |  |  |  |
| ASV933 Firmicutes; Clostridia; Lachnospirales; Lachnospiraceae | 0.310 |  |  |  |  |  |  |
| ASV1696 Firmicutes; Clostridia; Lachnospirales; Lachnospiraceae; Lachnoclostridium | 0.301 |  |  |  |  |  |  |
| ASV1640 Firmicutes; Clostridia; Lachnospirales; Lachnospiraceae | 0.276 |  |  |  |  |  |  |
| ASV3899 Proteobacteria; Gammaproteobacteria; Enterobacterales; Enterobacteriaceae; Klebsiella | 0.270 |  |  |  |  |  |  |
| ASV957 Firmicutes; Clostridia; Lachnospirales; Lachnospiraceae; Lachnospiraceae_NK4A136_group | 0.218 |  |  |  |  |  |  |
| ASV3526 Proteobacteria; Gammaproteobacteria; Enterobacterales; Enterobacteriaceae; Klebsiella | 0.204 |  |  |  |  |  |  |
| ASV3557 Actinobacteriota; Actinobacteria; Corynebacteriales; Corynebacteriaceae; Lawsonella | 0.190 |  |  |  |  |  |  |
| ASV1378 Firmicutes; Clostridia; Oscillospirales; Oscillospiraceae; UCG-002; uncultured_organism | 0.189 |  |  |  |  |  |  |
| ASV1634 Firmicutes; Clostridia; Oscillospirales; Ruminococcaceae | 0.185 |  |  |  |  |  |  |
| ASV1868 Firmicutes; Bacilli; Staphylococcales; Staphylococcaceae; Staphylococcus | 0.136 |  |  |  |  |  |  |
| ASV2457 Firmicutes; Negativicutes; Veillonellales-Selenomonadales; Veillonellaceae; Veillonella | 0.132 |  |  |  |  |  |  |
| ASV947 Firmicutes; Clostridia; Lachnospirales; Lachnospiraceae | 0.130 |  |  |  |  |  |  |
| ASV1396 Firmicutes; Clostridia; Lachnospirales; Lachnospiraceae; Lachnospiraceae_NK4A136 | 0.122 |  |  |  |  |  |  |
| ASV3988 Proteobacteria; Gammaproteobacteria; Enterobacterales; Enterobacteriaceae; Klebsiella | 0.108 |  |  |  |  |  |  |
| ASV3585 Firmicutes; Clostridia; Peptostreptococcales-Tissierellales; Ezakiella | 0.092 |  |  |  |  |  |  |
| ASV3184 Firmicutes; Negativicutes; Veillonellales-Selenomonadales; Veillonellaceae; Veillonella | 0.060 |  |  |  |  |  |  |
| ASV3722 Actinobacteriota; Actinobacteria; Bifidobacteriales; Bifidobacteriaceae; Bifidobacterium |  | 4.633 |  |  |  |  |  |
| ASV907 Firmicutes; Clostridia; Lachnospirales; Lachnospiraceae; Ruminococcus torquegroup |  | 3.450 |  |  |  |  |  |
| ASV619 Firmicutes; Clostridia; Lachnospirales; Lachnospiraceae; Blautia |  | 1.928 |  |  |  |  |  |
| ASV954 Firmicutes; Clostridia; Lachnospirales; Lachnospiraceae; Ruminococcus torquegroup |  | 1.749 |  |  |  |  |  |
| ASV1647 Proteobacteria; Gammaproteobacteria; Burkholderiales; Sutterellaceae; Sutterella |  | 1.599 |  |  |  |  |  |
| ASV143 Actinobacteriota; Actinobacteria; Bifidobacteriales; Bifidobacteriaceae; Bifidobacterium bifidum | | 1.243 |  |  |  |  |  |
| ASV428 Actinobacteriota; Actinobacteria; Bifidobacteriales; Bifidobacteriaceae; Bifidobacterium longum |  | 1.074 |  |  |  |  |  |
| ASV950 Firmicutes; Clostridia; Oscillospirales; Ruminococcaceae; Subdoligranulum |  | 0.576 |  |  |  |  |  |
| ASV741 Firmicutes; Clostridia; Lachnospirales; Lachnospiraceae; Blautia |  | 0.554 |  |  |  |  |  |
| ASV490 Firmicutes; Clostridia; Lachnospirales; Lachnospiraceae; Anaerostipes |  | 0.513 |  |  |  |  |  |
| ASV744 Firmicutes; Clostridia; Lachnospirales; Lachnospiraceae; Eubacterium hallii group |  | 0.273 |  |  |  |  |  |
| ASV1724 Firmicutes; Clostridia; Oscillospirales; Oscillospiraceae; UCG-002 |  | 0.272 |  |  |  |  |  |
| ASV1603 Firmicutes; Clostridia; Lachnospirales; Lachnospiraceae; Eubacterium hallii group |  | 0.240 |  |  |  |  |  |
| ASV1978 Actinobacteriota; Actinobacteria; Propionibacteriales; Propionibacteriaceae; Cutibacterium |  | 0.101 |  |  |  |  |  |
| ASV661 Firmicutes; Clostridia; Lachnospirales; Lachnospiraceae; Anaerostipes |  | 0.075 |  |  |  |  |  |
| ASV1020 Firmicutes; Bacilli; Lactobacillales; Lactobacillaceae; Lactobacillus |  | 0.053 |  |  |  |  |  |
| ASV3953 Firmicutes; Bacilli; Lactobacillales; Lactobacillaceae; Lactobacillus |  | 0.050 |  |  |  |  |  |
| ASV3631 Firmicutes; Bacilli; Lactobacillales; Lactobacillaceae; Lactobacillus |  | 0.038 |  |  |  |  |  |
| ASV1016 Bacteroidota; Bacteroidia; Bacteroidales; Tannerellaceae; Parabacteroides |  | 0.033 |  |  |  |  |  |
| ASV467 Bacteroidota; Bacteroidia; Bacteroidales; Bacteroidaceae; Bacteroides |  | 0.033 |  |  |  |  |  |
| ASV46 Firmicutes; Negativicutes; Selenomonadaceae; Megamonas; Megamonafuniformis |  |  | 4.421 |  |  |  |  |
| ASV2583 Bacteroidota; Bacteroidia; Bacteroidales; Bacteroidaceae; Bacteroides; Bacteroidefragilis |  |  | 2.852 |  |  |  |  |
| ASV1628 Firmicutes; Negativicutes; Veillonellales-Selenomonadales; Selenomonadaceae; Megamonas |  |  | 2.734 |  |  |  |  |
| ASV2975 Firmicutes; Negativicutes; Veillonellales-Selenomonadales; Selenomonadaceae; Megamonas |  |  | 1.282 |  |  |  |  |
| ASV347 Bacteroidota; Bacteroidia; Bacteroidales; Prevotellaceae; Prevotella; Prevotella_intermedia |  |  | 1.224 |  |  |  |  |
| ASV2232 Firmicutes; Clostridia; Peptostreptococcales-Tissierellales; Peptostreptococcaceae; Peptostreptococcus | |  | 0.301 |  |  |  |  |
| ASV3187 Firmicutes; Bacilli; Erysipelotrichales; Erysipelotrichaceae; Solobacterium; uncultured_organism |  |  |  | 0.001 |  |  |  |
| ASV3540 Fusobacteriota; Fusobacteriia; Fusobacteriales; Fusobacteriaceae; Fusobacterium |  |  |  | 6.237 |  |  |  |
| ASV3959 Firmicutes; Bacilli; Lactobacillales; Streptococcaceae; Streptococcus; Streptococcusalivarius |  |  |  | 0.765 |  |  |  |
| ASV3538 Bacteroidota; Bacteroidia; Bacteroidales; Porphyromonadaceae; Porphyromonas |  |  |  |  | 4.590 |  |  |
| ASV2131 Firmicutes; Clostridia; Lachnospirales; Lachnospiraceae; Ruminococcus torquegroup |  |  |  |  | 3.067 |  |  |
| ASV2618 Firmicutes; Bacilli; Erysipelotrichales; Erysipelotrichaceae; Solobacterium |  |  |  |  | 1.881 |  |  |
| ASV2484 Firmicutes; Clostridia; Oscillospirales; Ruminococcaceae; UBA1819; uncultured_organism |  |  |  |  | 1.437 |  |  |
| ASV3234 Firmicutes; Negativicutes; Veillonellaceae; Dialister; Dialister pneumosintes |  |  |  |  | 0.965 |  |  |
| ASV2577 Bacteroidota; Bacteroidia; Bacteroidales; Bacteroidaceae; Bacteroides |  |  |  |  | 0.867 |  |  |
| ASV783 Firmicutes; Clostridia; Lachnospirales; Lachnospiraceae |  |  |  |  | 0.621 |  |  |
| ASV3288 Fusobacteriota; Fusobacteriia; Fusobacteriales; Fusobacteriaceae; Fusobacterium |  |  |  |  | 0.441 |  |  |
| ASV1694 Firmicutes; Clostridia; Lachnospirales; Lachnospiraceae; Lachnospiraceae_UCG-010 |  |  |  |  | 0.386 |  |  |
| ASV2107 Firmicutes; Clostridia; Lachnospirales; Lachnospiraceae; Lachnoclostridium |  |  |  |  | 0.383 |  |  |
| ASV407 Firmicutes; Clostridia; Lachnospirales; Lachnospiraceae; Hungatella |  |  |  |  | 0.355 |  |  |
| ASV3069 Firmicutes; Clostridia; Peptostreptococcales-Tissierellales; Anaerovoracaceae | | | |  | 0.323 |  | 0.323 |
| ASV1638 Firmicutes; Clostridia; Oscillospirales; Oscillospiraceae; UCG-002; uncultured_rumen |  |  |  |  | 0.291 |  |  |
| ASV1415 Bacteroidota; Bacteroidia; Bacteroidales; Bacteroidaceae; Bacteroides |  |  |  |  | 0.148 |  |  |
| ASV1368 Firmicutes; Clostridia; Lachnospirales; Lachnospiraceae; Lachnospiraceae UCG-004 |  |  |  |  | 0.108 |  |  |
| ASV1777 Firmicutes; Clostridia; Lachnospirales; Lachnospiraceae; Hungatella |  |  |  |  | 0.066 |  |  |
| ASV399 Firmicutes; Clostridia; Christensenellales; Christensenellaceae; Catabacter hongkongensis | |  |  |  | 0.023 |  |  |
| ASV500 Firmicutes; Bacilli; Erysipelotrichales; Erysipelotrichaceae; Holdemania |  |  |  |  | 0.019 |  |  |
| ASV2254 Firmicutes; Clostridia; Lachnospirales; Lachnospiraceae; Eubacterium fissicatena |  |  |  |  | 0.015 |  |  |

**Table S4** Summary of NESH score and Jaccard score for driver nodes between benign polyps and colorectal cancer stage 1 based on NetShift analysis

| ASV ID and taxonomy | Jaccard-score | NESH-score |
| --- | --- | --- |
| ASV2473 *Phascolarctobacterium succinatutens* | 0.00 | 2.89 |
| ASV3703 *Muribaculum intestinale* | 0.00 | 2.89 |
| ASV853 *Neglectibacter timonensis* | 0.00 | 2.80 |
| ASV3538 *Porphyromonas endodontalis* | 0.00 | 2.61 |
| ASV1531 *Subdoligranulum variabile* | 0.07 | 2.30 |
| ASV1839 *Clostridium saudiense* | 0.00 | 2.13 |
| ASV1014 *Faecalibacterium prausnitzii* | 0.15 | 2.06 |
| ASV2467 *Parabacteroides distasonis* | 0.00 | 1.92 |
| ASV1414 *Bifidobacterium stercoris* | 0.00 | 1.92 |
| ASV78 *Odoribacter splanchnicus* | 0.00 | 1.80 |
| ASV2131 *Ruminococcus torques* | 0.00 | 1.75 |
| ASV921 *Coprococcus comes* | 0.08 | 1.13 |
| ASV963 *Dorea formicigenerans* | 0.18 | 1.01 |

**Table S5** The predicted accuracy based on profiles of the top 19 colorectal cancer (CRC) stage-discriminatory ASVs (data not shown) without removal of the nine age-discriminatory ASVs (Fig. S6). For a given sample, the consistency between observed and predicted category was termed as a correct diagnosis, otherwise it was termed as a false diagnosis. Bold values represent the numbers of correct diagnoses.

| Observed status | Predicted health status | | | | | Overall accuracy |
| --- | --- | --- | --- | --- | --- | --- |
|  | T1 | T2 | T3 | T4 | BP |  |
| T1 | **4** | 1 | 2 | 0 | 2 | (68/95) 71.6% |
| T2 | 0 | **2** | 10 | 0 | 1 |  |
| T3 | 1 | 2 | **16** | 0 | 3 |  |
| T4 | 0 | 0 | 2 | **4** | 0 |  |
| BP | 0 | 0 | 3 | 0 | **42** |  |

**Figure S1** Comparison of carcinoembryonic antigen (CEA) (A) and body mass index (BMI) (B) along the four colorectal cancer stages, T1, T2, T3 and T4.


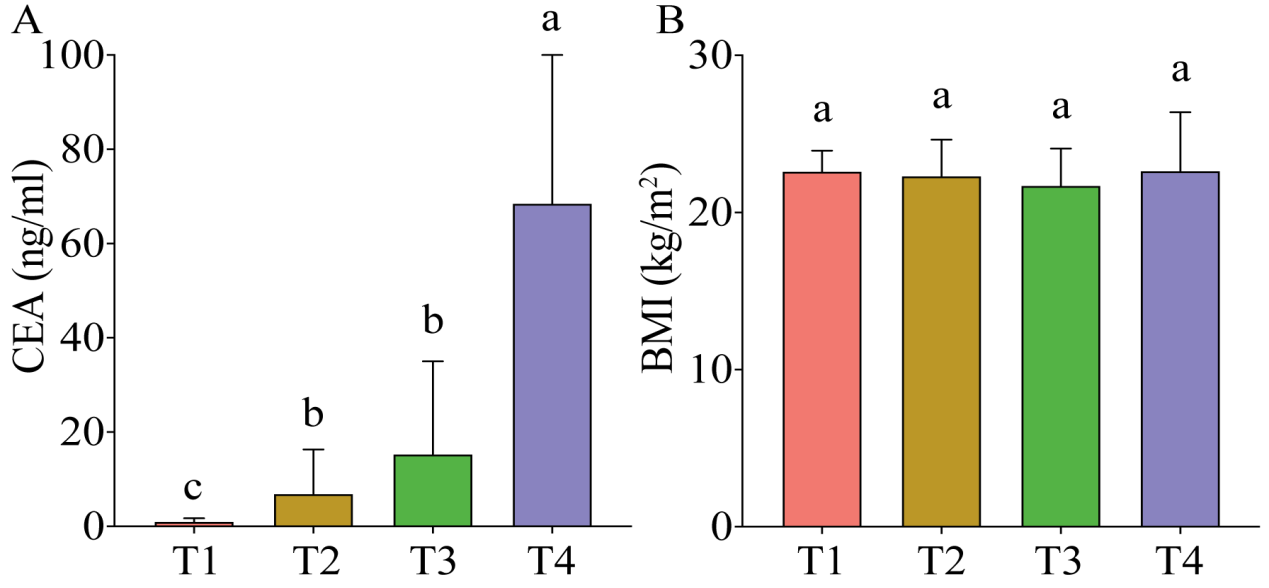


**Figure S2** The rarefaction curves of each sample based on observed species.

**
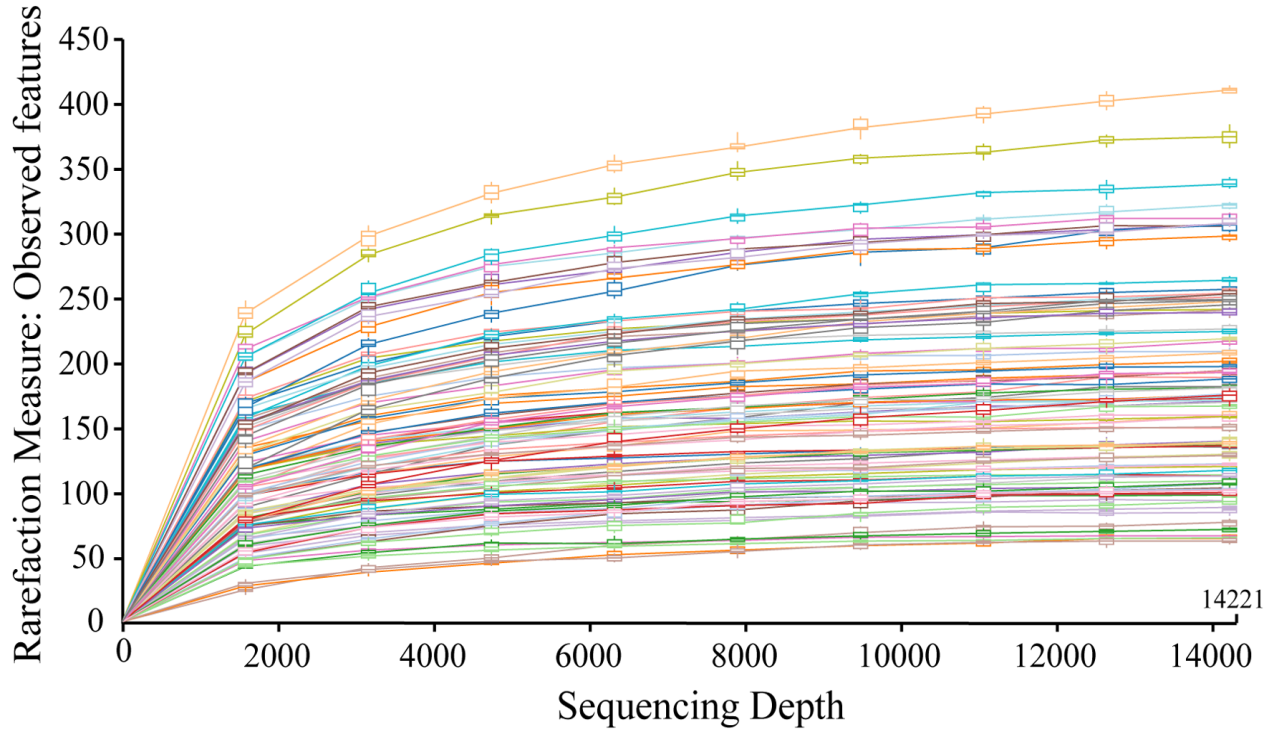
**

**Figure S3** Comparing the relative abundances of dominant bacterial phyla (A) and genera (B) in benign polyps (BP) and along the four colorectal cancer stages, T1, T2, T3 and T4 using one way-analysis of variance (ANOVA).

**
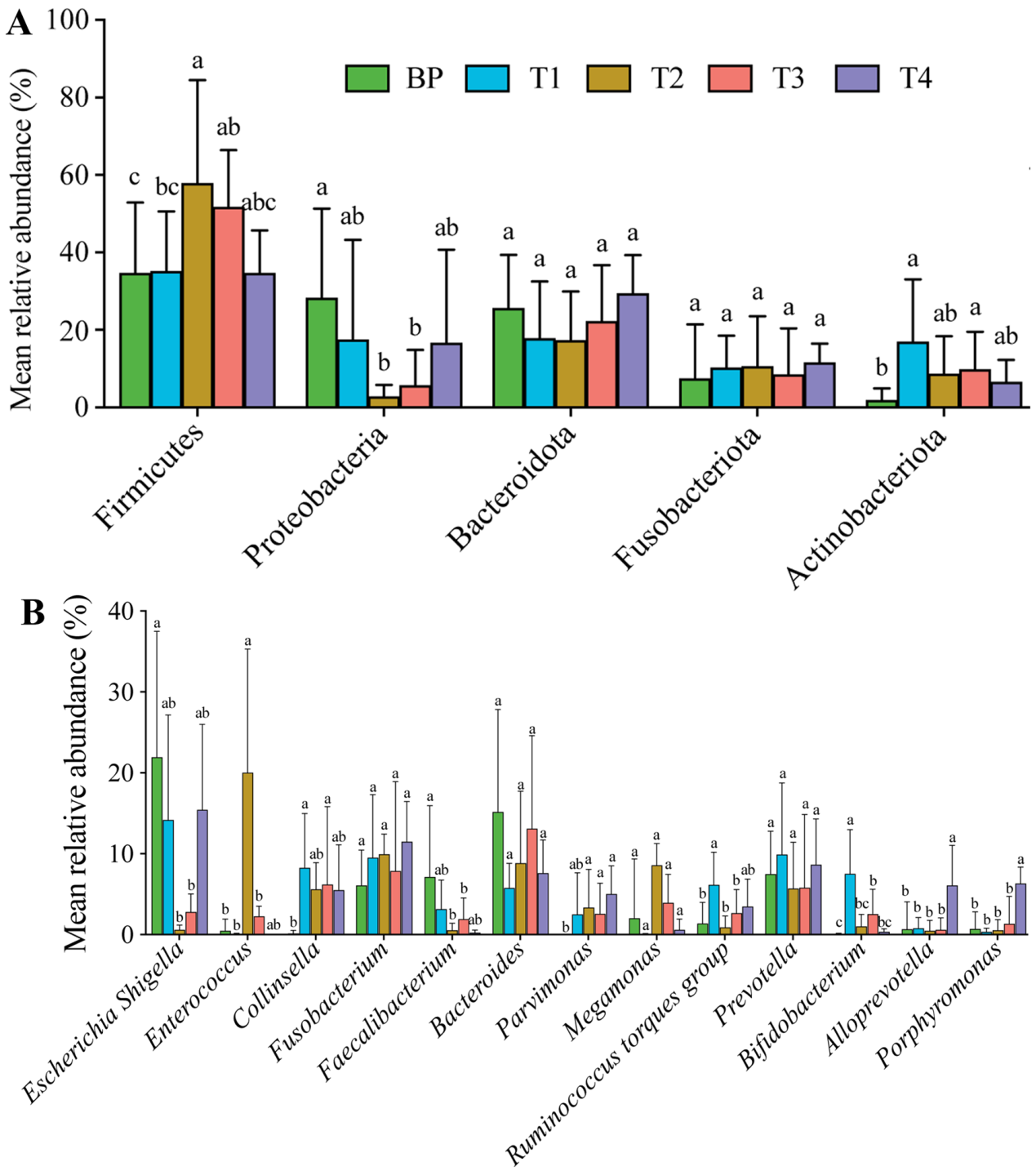
**

**Figure S4** Comparing Shannon index (A) and Phylogenetical diversity (B) of bacterial communities in benign polyps (BP) and along the four colorectal cancer stages, T1, T2, T3 and T4.


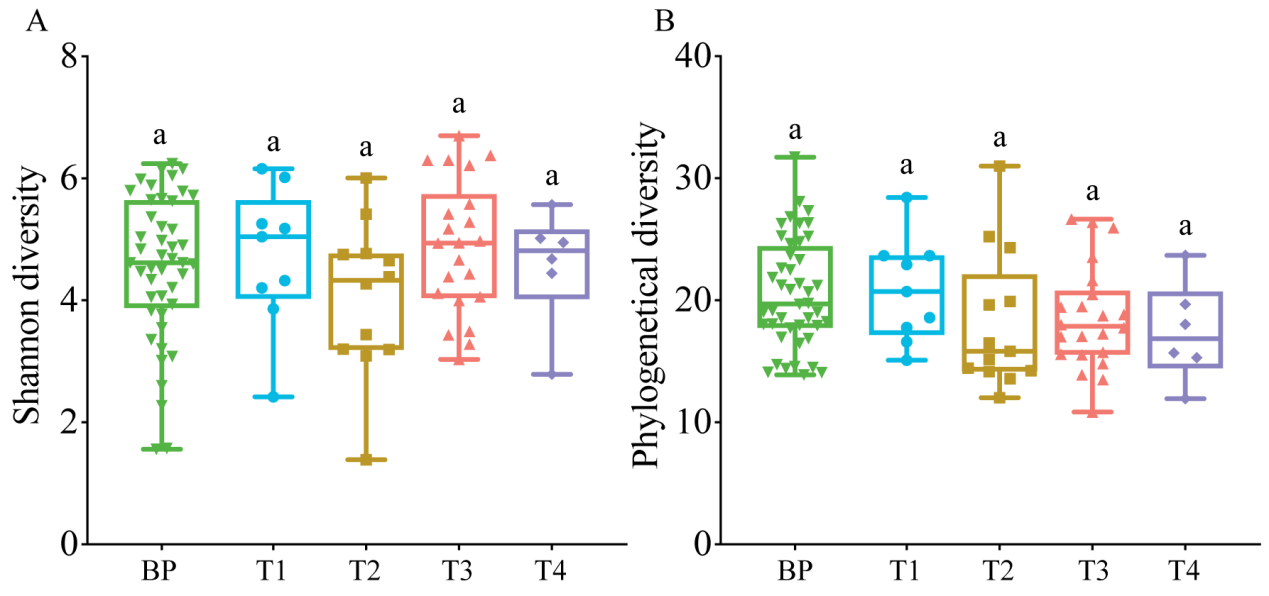


**Figure S5** Heatmaps showing the ASVs that were the most abundant in T1 (A) or T4 (B) stage compared with benign polyps (BP) and along the four colorectal cancer stages.


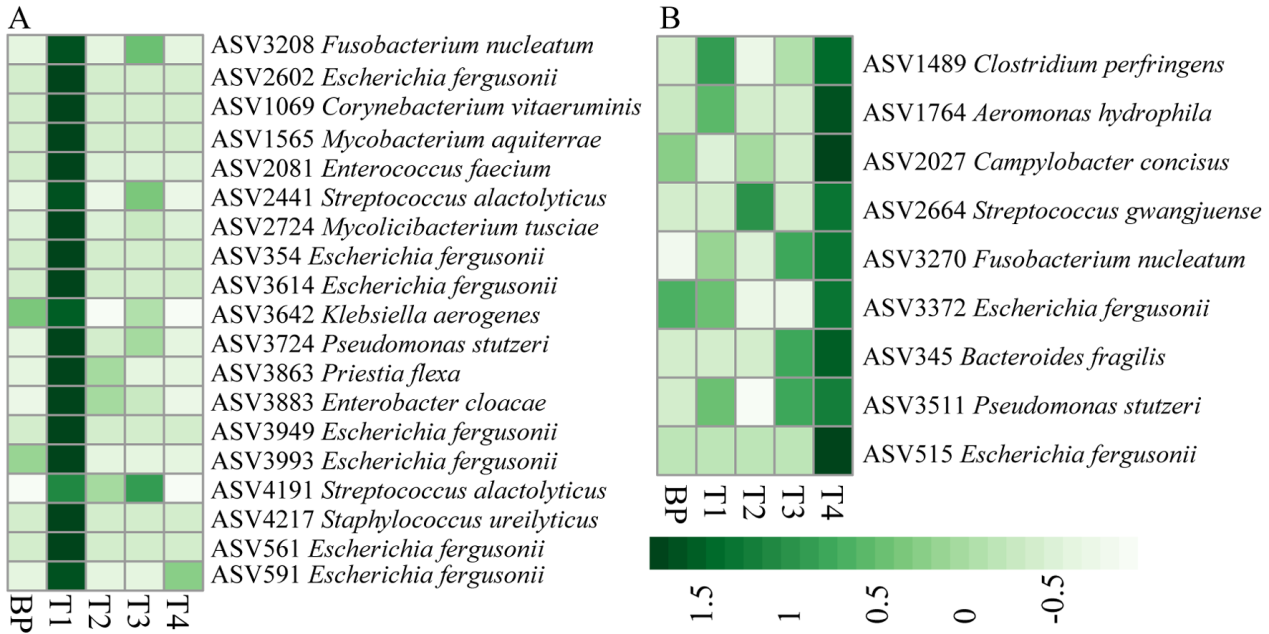


**Figure S6** Age-discriminatory ASVs (amplicon sequence variants) for defining host age. The top nine age-discriminatory ASVs were identified by regression of their relative abundances against chronologic age in the 45 benign polyps using 10-fold cross-validation (A). and their taxnomic information (B). The consistency (Pearson *r* = 0.924, *P* < 0.001) between predicted and chronologic ages acorss the 45 healthy contorls (C).


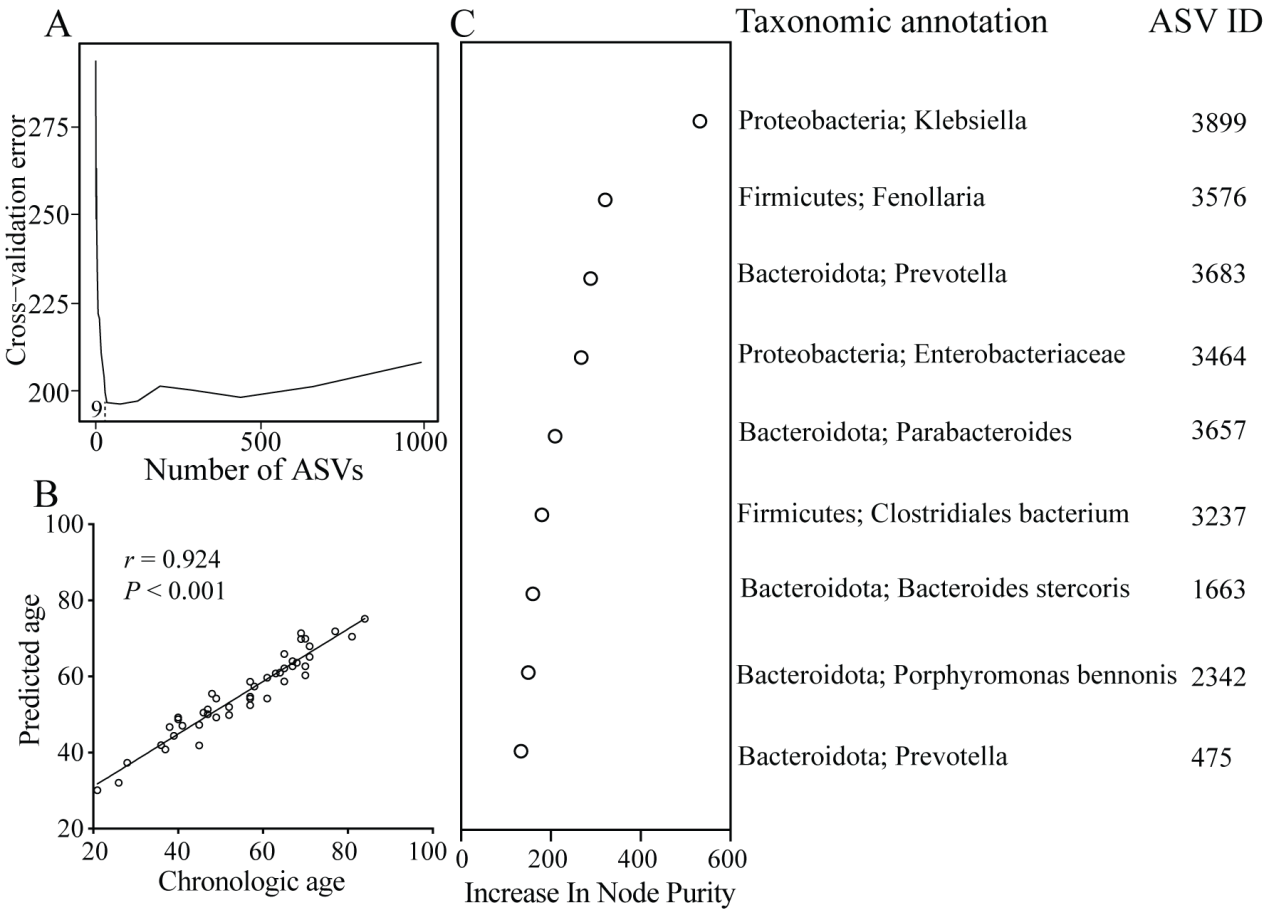


**Figure S7** Phylogenetic tree of the 15 CRC stage-discriminatory taxa and their closest relatives. The same color represents the same phyla level.


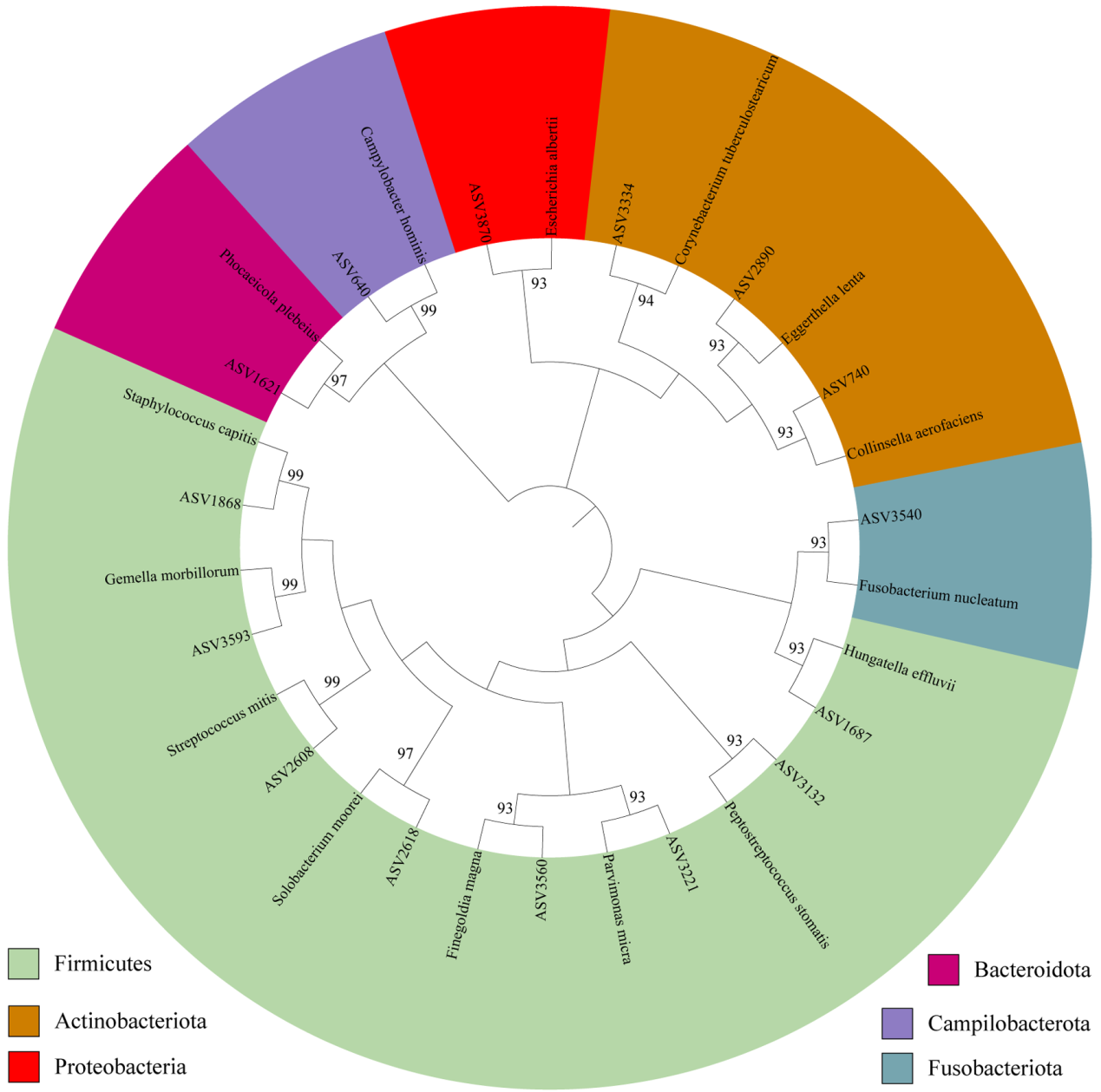


**Figure S8** The diagnosed CRC stages using profiles of the 15 CRC stage-discriminatory ASVs according to gender (A) and age (B). The consistency between observed and diagnosed stage was termed a correct diagnosis with a cutoff of 50%.


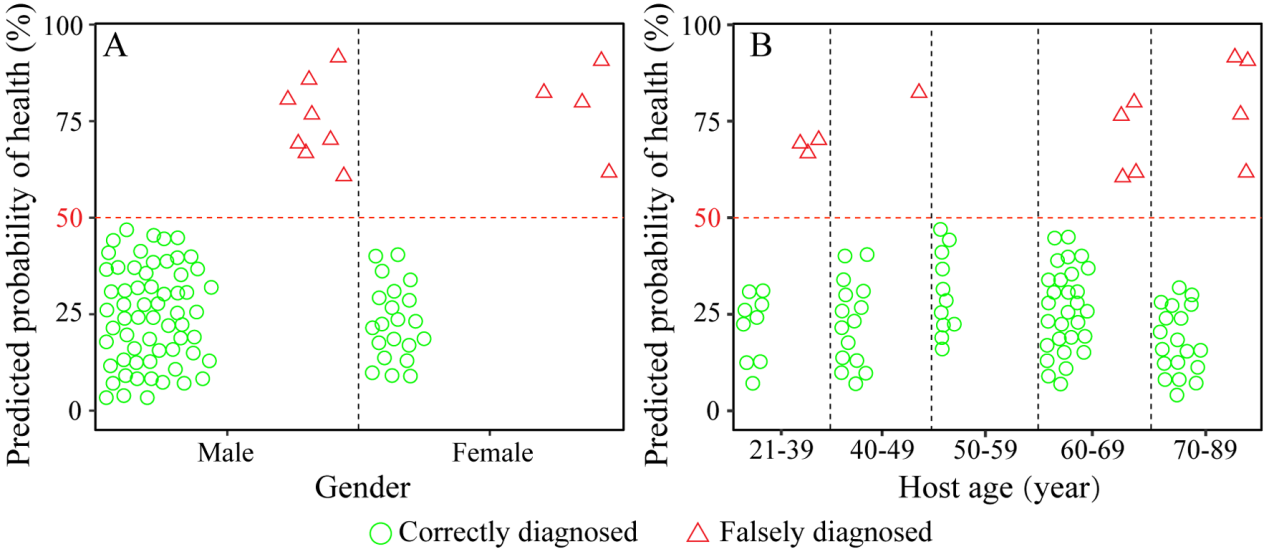

Supplement: Supplementary file 1 [file DataSheet_1.docx]
